# Cortical Thickness Abnormalities at Different Stages of the Illness Course in Schizophrenia: A Systematic Review and Meta-analysis

**DOI:** 10.1001/jamapsychiatry.2022.0799

**Published:** 2022-04-27

**Authors:** Youjin Zhao, Qian Zhang, Chandan Shah, Qian Li, John A. Sweeney, Fei Li, Qiyong Gong

**Affiliations:** 1 Huaxi MR Research Center, Department of Radiology, West China Hospital of Sichuan University, Chengdu, Sichuan, China; 2 Research Unit of Psychoradiology, Chinese Academy of Medical Sciences, Chengdu, Sichuan, China; 3 Department of Psychiatry and Behavioral Neuroscience, University of Cincinnati, Cincinnati, Ohio; 4 Department of Radiology, West China Xiamen Hospital of Sichuan University, Xiamen, Fujian, China

## Abstract

**Importance:**

Questions of whether and how cortical thickness (CTh) alterations differ over the course of schizophrenia (SCZ) have yet to be resolved.

**Objective:**

To characterize CTh alterations across illness stages in SCZ.

**Data Sources:**

PubMed, Embase, Web of Science, and Science Direct were screened for CTh studies published before June 15, 2021.

**Study Selection:**

Original studies comparing whole-brain CTh alterations from healthy controls in individuals at clinical high-risk (CHR), first episode of psychosis (FEP), and long-term illness stages of SCZ were included.

**Data Extraction and Synthesis:**

This preregistered systematic review and meta-analysis followed PRISMA reporting guidelines. Separate and pooled meta-analyses were performed using seed-based d mapping. Meta-regression analyses were conducted.

**Main Outcomes and Measures:**

Cortical thickness differences from healthy control individuals across illness stages.

**Results:**

Ten studies comprising 859 individuals with CHR (mean [SD] age, 21.02 [2.66] years; male, 573 [66.7%]), 12 studies including 671 individuals with FEP (mean [SD] age, 22.87 [3.99] years; male, 439 [65.4%]), and 10 studies comprising 579 individuals with long-term SCZ (mean [SD] age, 41.58 [6.95] years; male, 396 [68.4%]) were included. Compared with healthy control individuals, individuals with CHR showed cortical thinning in bilateral medial prefrontal cortex (*z* = −1.01; *P* < .001). Individuals with FEP showed cortical thinning in right lateral superior temporal cortex (*z* = −1.34; *P* < .001), right anterior cingulate cortex (*z* = −1.44; *P* < .001), and right insula (*z* = −1.14; *P* = .002). Individuals with long-term SCZ demonstrated CTh reductions in right insula (*z* = −3.25; *P* < .001), right inferior frontal cortex (*z* = −2.19; *P* < .001), and left (*z* = −2.37; *P* < .001) and right (*z* = −1.94; *P* = .002) temporal pole. There were no significant CTh differences between CHR and FEP. Individuals with long-term SCZ showed greater cortical thinning in right insula (*z* = −2.58; *P* < .001), right inferior frontal cortex (*z* = −2.32; *P* < .001), left lateral temporal cortex (*z* = −1.91; *P* = .002), and right temporal pole (*z* = −1.82; *P* = .002) than individuals with FEP. Combining all studies on SCZ, accelerated age-related CTh reductions were found in bilateral lateral middle temporal cortex and right pars orbitalis in inferior frontal cortex.

**Conclusions and Relevance:**

The absence of significant differences between FEP and CHR noted in this systematic review and meta-analysis suggests that the onset of psychosis was not associated with robust CTh reduction. The greater cortical thinning in long-term SCZ compared with FEP with accelerated age-related reduction in CTh suggests progressive neuroanatomic alterations following illness onset. Caution in interpretation is needed because heterogeneity in samples and antipsychotic treatment may confound these results.

## Key Points

**Question:**

Are there differences in cortical thickness (CTh) alterations between clinical high-risk (CHR), first episode of psychosis (FEP), and long-term illness stages of schizophrenia (SCZ)?

**Findings:**

In meta-analyses comprising 2109 individuals across different illness stages of SCZ (10 studies of CHR, 12 studies of FEP, and 10 studies of long-term SCZ), CTh did not differ significantly between individuals with CHR and FEP, but those with long-term illness showed more pronounced CTh reductions than individuals with FEP. Accelerated age-related CTh reductions were found in frontotemporal cortex when combining all studies.

**Meaning:**

The findings of this systematic review and meta-analysis do not indicate an emergence of CTh alterations with illness onset but suggest a progressively increasing thinning of CTh after illness onset.

## Teaser

This systematic review and meta-analysis examines changes in cortical thickness in individuals during differing phases across the course of schizophrenia.

## Introduction

Schizophrenia (SCZ) is a debilitating psychotic disorder typically beginning in late adolescence or early adulthood, often followed by recurrent episodes of psychosis and, in some cases, declining psychosocial function.^1^ The onset of illness is usually preceded by a clinical high-risk (CHR) phase characterized by attenuated psychosis symptoms, brief limited psychotic symptoms, and functional deterioration.^2^ Potential brain changes between CHR and onset of the first episode of psychosis (FEP) that might reflect neurotoxicity of acute psychosis, and between FEP and long-term illness that might reflect progressive brain abnormalities, are important issues being addressed in several SCZ research programs.

Previous neuroimaging studies have documented widespread structural brain alterations at different stages of SCZ.^3,4^ However, because most studies focused on only 1 specific illness stage, questions of how these alterations may differ over the illness course have yet to be fully resolved. Few longitudinal studies have been completed with currently available image analysis pipelines, and few studies have compared patients with CHR and FEP.^3^ Some cross-sectional studies of patients after illness onset have identified an accelerated rate of age-related decline in brain features compared with healthy individuals, which provides preliminary evidence for progressive anatomic changes over the illness course.^5,6,7,8^ A systematic review and meta-analysis comparing individuals at different illness stages might characterize brain abnormalities over the illness course with robustness enhanced by combining data across the published literature.

Clinically, there are 2 broad issues of particular interest. The first is whether there are greater brain abnormalities in individuals with FEP than CHR. This issue is related to questions about whether the onset of acute psychosis itself induces brain alterations. The second issue is whether, following onset of the illness, there are increasing brain alterations that might be targets for therapeutic intervention. Addressing these issues is also important from the perspective of brain imaging biomarker development.

Recently, regional cortical thickness (CTh) measurement via surface-based morphometry has been used to evaluate brain anatomy in patients with SCZ.^9^ Cortical thickness analysis can better reflect underlying pathophysiologic mechanisms than gray matter volume analysis.^10^ Cortical thickness alterations have been identified in SCZ, but the regional distribution of detected abnormalities has not been consistent, even within studies of specific phases of illness.^11^ Individuals with CHR have demonstrated varying CTh alterations,^12,13,14^ with those who subsequently developed psychosis having greater abnormalities.^15,16,17^ Cortical thinning has been reported in patients with FEP in prefrontal,^18^ lateral temporal,^19,20^ and parietal cortex,^21^ albeit with negative findings.^21,22^ Discrepancies might be related to small sample sizes, variable sample characteristics, medication status, and analytical methods.

Few systematic review and meta-analysis studies have examined CTh, with most considering volume measures (eTable 1 in the Supplement),^23,24^ and none compared thickness across the 3 illness stages. Findings from earlier studies include a smaller parahippocampal volume in long-term SCZ than FEP.^25^ Both individuals with CHR and those with FEP have shown similar cortical thickening in bilateral occipital regions.^13^ Individuals with FEP exhibited thinner right posterior cingulate cortex than those with CHR.^20^ More widespread and pronounced CTh reductions were found in individuals with long-term SCZ than CHR and FEP.^26^ A limited number of longitudinal studies have found progressive CTh reductions in long-term SCZ, predominantly in frontotemporal regions.^3^ These findings generally parallel observations from neurophysiologic and neuropsychological studies.^27,28^

Given the clinical importance of understanding the course of illness in SCZ and the variability in findings across single studies, we conducted coordinate-based meta-analyses of published studies on CTh in individuals with CHR, FEP, and long-term SCZ, using seed-based d mapping software. We performed case-control meta-analyses in each illness phase, followed by quantitative comparisons of findings between illness stages. Our primary interest was testing for increases in abnormalities in FEP compared with CHR samples and in long-term SCZ compared with FEP samples. We tested for associations between clinical and demographic variables with anatomic alterations, using meta-regression analyses.

## Methods

### Search Strategy and Selection Criteria

Systematic review and meta-analysis was carried out using the Preferred Reporting Items for Systematic Reviews and Meta-analyses (PRISMA) reporting guideline.^29^ The protocol was registered on PROSPERO (CRD42021253875). Details of the search strategy and selection criteria are provided in the eMethods in the Supplement. Two of us (Y.Z. and Q.Z.) independently conducted literature searches in PubMed, Embase, Web of Science, and Science Direct and screened the results for CTh studies published before June 15, 2021. Inconsistencies were discussed and a consensus was reached.

### Meta-analysis

Meta-analyses of CTh studies were conducted using seed-based d mapping software (SDM, version 5.15, SDM Project). First, coordinates of cluster peaks and effect sizes of significant between-group differences were used to create an effect-size signed map for each study. Negative findings were included and estimated conservatively to have a null effect size. Next, a random-effects analysis was performed to obtain the mean map, combining data of each included study with both positive and negative differences in the same map.^30^

Separate analyses were performed to identify CTh alterations at each illness stage vs healthy control individuals. We then performed a pooled meta-analysis comparing patients at different illness stages using randomization tests to identify significant differences and a regression analysis across all samples to test for correlations with demographic and clinical features. Quadratic models of age effects were used when significantly better than linear models. Details of quality assessment and data recording, seed-based d mapping method of meta-analysis, jackknife, heterogeneity, and publication bias analysis, and meta-regression analysis are presented in the eMethods in the Supplement. All tests were 2-sided and unpaired, and a statistical threshold of *P* < .005 was used with a cluster extent of 10 voxels for the meta-analysis.

## Results

### Included Studies and Sample Characteristics

**Figure 1.**
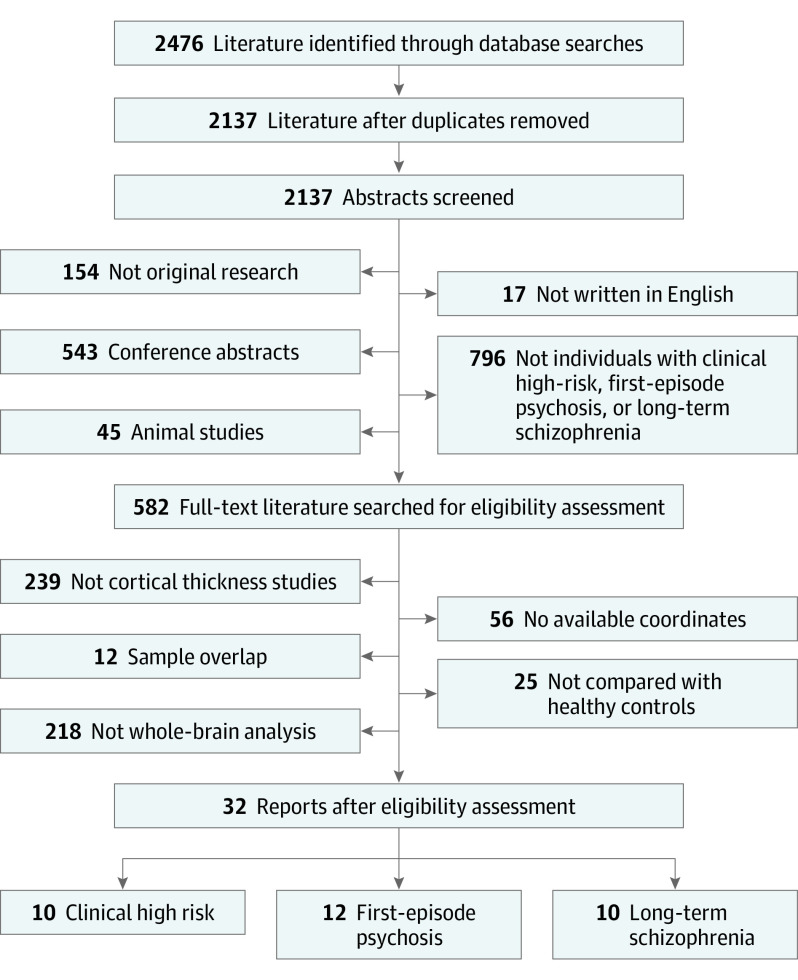
Flowchart of Literature Search and Selection Criteria

Figure 1 shows the PRISMA flowchart of the literature search and eligibility assessment. Ten studies^12,13,14,15,16,17,31,32,33,34^ comprising 859 individuals with CHR (mean [SD] age, 21.02 [2.66] years; male, 573 [66.7%]; female, 286 [33.3%]) and 531 healthy control individuals (mean [SD] age, 21.67 [3.03] years; male, 300 [56.5%]; female, 231 [43.5%]), 12 studies^13,18,19,21,22,35,36,37,38,39,40,41^ including 671 individuals with FEP (mean [SD] age, 22.87 [3.99] years; male, 439 [65.4%]; female, 232 [34.6%]) and 566 healthy control individuals (mean [SD] age, 23.39 [4.5] years; male, 349 [61.7%]; female, 217 [38.3%]), and 10 studies^5,11,42,43,44,45,46,47,48,49^ comprising 579 individuals with long-term SCZ (mean [SD] age, 41.58 [6.95] years; male, 396 [68.4%]; female, 183 [31.6%]) and 475 healthy control individuals (mean [SD] age, 40.07 [7.0] years; male, 303 [63.8%]; female, 172 [36.2%]) were included in the meta-analysis. Four of 12 FEP studies included some patients with a current diagnosis of affective psychosis (proportion, 9.7%). For longitudinal studies, only baseline data were included to avoid bias toward the outcomes of the interventions or illness progression. Results of quality assessments (eResults and eTable 2 in the Supplement) and detailed characteristics of included studies (eResults and eTable 3 in the Supplement) are provided.

### Meta-analysis and Regression in Each Group

**Figure 2.**
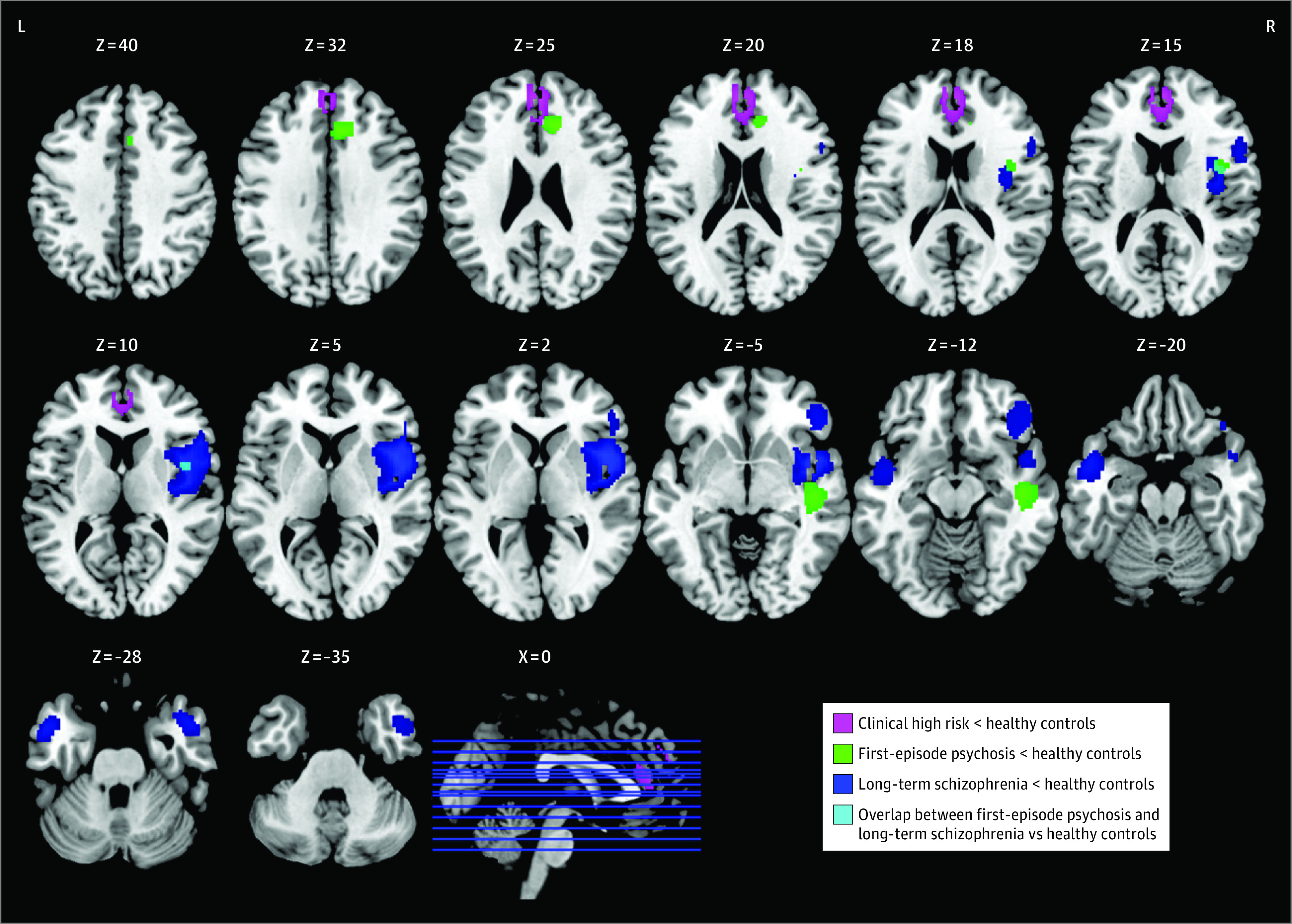
Cortical Thickness Reductions Within Studies of Each of the 3 Illness Stages Compared with healthy controls, clinical high-risk individuals showed cortical thinning in the bilateral medial prefrontal cortex extending to bilateral superior frontal cortex and anterior cingulate cortex. Individuals with first episode of psychosis showed cortical thinning in the right lateral superior temporal cortex, right anterior cingulate cortex, and right insula; individuals with long-term schizophrenia showed cortical thinning in the right insula extending to frontal operculum, right pars orbitalis of inferior frontal cortex, and more widespread cortical thickness reductions in temporal cortex. The horizontal blue lines indicate the location of these axial images. L indicates left; R, right.

Compared with healthy control individuals, individuals with CHR showed cortical thinning of 1 cluster in bilateral medial prefrontal cortex (*z* = −1.01; *P* < .001) extending to bilateral superior frontal cortex, bilateral anterior cingulate cortex, and right middle cingulate cortex (Figure 2, Table; eFigure 1 in the Supplement). No significant association of age was found in this cluster (eTable 4 in the Supplement).

**Table.** Results of the Present Meta-analyses

Comparison	Peak region	MNI coordinate			SDM, *z* score	*P* value, uncorrected	Voxels	Cluster breakdown and full region of cluster (No. of voxels)
		x	y	z				
CHR<healthy control individuals	Left medial prefrontal cortex	−4	50	24	−1.01	<.001	598	Left superior frontal cortex, medial (218)
								Right anterior cingulate cortex (165)
								Left anterior cingulate cortex (147)
								Right superior frontal cortex, medial (65)
								Right middle cingulate cortex (3)
FEP<healthy control individuals	Right lateral superior temporal cortex	44	−18	−8	−1.34	<.001	451	Right superior temporal cortex (307)
								Right middle temporal cortex (84)
								Right inferior temporal cortex (60)
	Right anterior cingulate cortex	8	28	30	−1.44	<.001	321	Right middle cingulate cortex (219)
								Right anterior cingulate cortex (102)
	Right insula	40	2	14	−1.14	.002	77	Right insula (42)
								Right superior temporal cortex (23)
								Right rolandic operculum (12)
Long-term SCZ<healthy control individuals	Right insula	46	8	4	−3.25	<.001	1336	Right insula (863)
								Right rolandic operculum (342)
								Right lenticular nucleus, putamen (131)
	Right inferior frontal cortex, orbital part	48	28	−4	−2.19	<.001	991	Right inferior frontal cortex, orbital part (462)
								Right inferior frontal cortex, opercular part (354)
								Right inferior frontal cortex, triangular part (175)
	Left temporal pole, middle temporal cortex	−50	4	−24	−2.37	<.001	730	Left temporal pole, middle temporal cortex (403)
								Left temporal pole, superior temporal cortex (200)
								Left inferior temporal cortex (127)
	Right temporal pole, superior temporal cortex	48	8	−18	−1.94	.002	915	Right temporal pole, superior temporal cortex (429)
								Right inferior temporal cortex (290)
								Right temporal pole, middle temporal cortex (196)
Pooled group<healthy control individuals	Right insula	44	4	4	−2.58	<.001	2585	Right insula (997)
								Right rolandic operculum (606)
								Right inferior frontal cortex, opercular part (354)
								Right temporal pole, superior temporal cortex (320)
								Right lenticular nucleus, putamen (166)
								Right Heschl’s gyrus (75)
								Right inferior frontal cortex, triangular part (67)
	Left anterior cingulate cortex	0	24	32	−1.71	.001	414	Right middle cingulate cortex (148)
								Left anterior cingulate cortex (91)
								Left middle cingulate cortex (83)
								Right anterior cingulate cortex (55)
								Left superior frontal cortex (22)
								Right superior frontal cortex (15)
	Right inferior frontal cortex, orbital part	46	34	−12	−1.75	<.001	277	Right inferior frontal cortex, orbital part (253)
								Right inferior frontal cortex, triangular part (24)
	Left lateral middle temporal cortex	−50	0	−20	−1.45	.003	63	Left middle temporal cortex (49)
								Left superior temporal cortex (14)
	Right lateral middle temporal cortex	48	6	−32	−1.42	.004	27	Right middle temporal cortex (27)
Long-term SCZ<FEP	Right insula	40	4	2	−2.58	<.001	981	Right insula (540)
								Right inferior frontal gyrus, opercular part (170)
								Right lenticular nucleus, putamen (129)
								Right rolandic operculum (109)
								Right superior temporal gyrus (19)
								Right inferior frontal gyrus, triangular part (14)
	Right inferior frontal cortex, orbital part	48	32	−12	−2.32	<.001	407	Right inferior frontal cortex, orbital part (341)
								Right inferior frontal cortex, triangular part (66)
	Left lateral middle temporal cortex	−50	0	−20	−1.91	.002	348	Left middle temporal cortex (214)
								Left superior temporal cortex (84)
								Left inferior temporal cortex (50)
	Right temporal pole, middle temporal cortex	50	6	−32	−1.82	.002	180	Right temporal pole, middle temporal cortex (125)
								Right inferior temporal cortex (36)
								Right temporal pole, superior temporal cortex (19)

Abbreviations: CHR, clinical high-risk; FEP, first episode of psychosis; MNI, Montreal Neurological Institute; SCZ, schizophrenia; SDM, seed-based d mapping.

Individuals with FEP showed CTh reductions in 3 clusters: right lateral superior temporal cortex (*z* = −1.34; *P*<.001), right anterior cingulate cortex (*z* = −1.44; *P* < .001), and right insula (*z* = −1.14; *P* = .002) (Figure 2, Table; eFigure 2 in the Supplement). No significant association of age or illness duration was found in any cluster (eTable 4 in the Supplement).

Individuals with long-term SCZ showed thinner cortex than healthy control individuals in 4 clusters: right insula extending to frontal operculum (*z* = −2.58; *P* < .001), right pars orbitalis of inferior frontal cortex (*z* = −2.32; *P* < .001), left lateral temporal cortex (*z* = −1.91; *P* = .002), and right temporal pole (*z* = −1.82; *P* = .002) (Figure 2, Table; eFigure 3 in the Supplement). In individuals with long-term SCZ, illness duration was associated with greater CTh reductions only in left anterior temporal cortex (*R*^2^ = 0.60; *P* = .003) (eFigure 4, eTable 4 in the Supplement).

### Comparisons of Illness Stages

**Figure 3.**
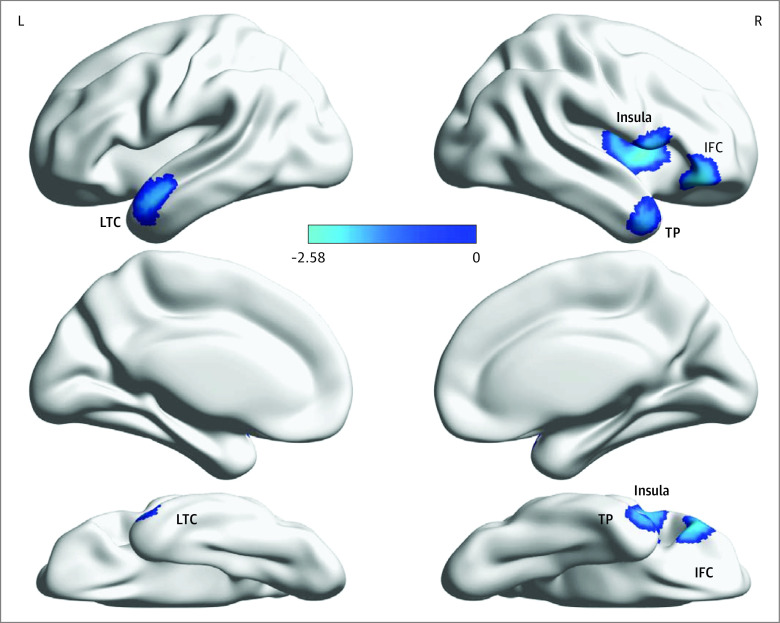
Cortical Thickness Differences Between First Episode of Psychosis and Long-term Schizophrenia Groups Compared with individuals with first episode of psychosis, those with long-term schizophrenia showed greater cortical thinning (cool color) in the right insula, right pars orbitalis of inferior frontal cortex (IFC), left lateral temporal cortex (LTC) (including superior temporal cortex and middle temporal cortex), and right temporal pole (TP). Color bar shows the seed-based d mapping *z* values. L indicates left; R, right.

We found no significant CTh differences between the CHR and FEP groups. Individuals with long-term SCZ showed greater cortical thickness reductions in right insula, right pars orbitalis of inferior frontal cortex, left lateral temporal cortex (including superior temporal cortex and middle temporal cortex [MTC]), and right temporal pole than those with FEP (Figure 3). Similar CTh reductions were seen in individuals with long-term SCZ compared with CHR (eFigure 5, eTable 5 in the Supplement).

### Pooled Meta-analysis and Regression

The combined group (all 3 illness stages) showed cortical thinning in 5 clusters, including right insula extending to frontal operculum, left anterior cingulate cortex extending to bilateral middle cingulate cortex, right pars orbitalis of inferior frontal cortex, and bilateral lateral MTC (eFigure 6 in the Supplement; Table).

**Figure 4.**
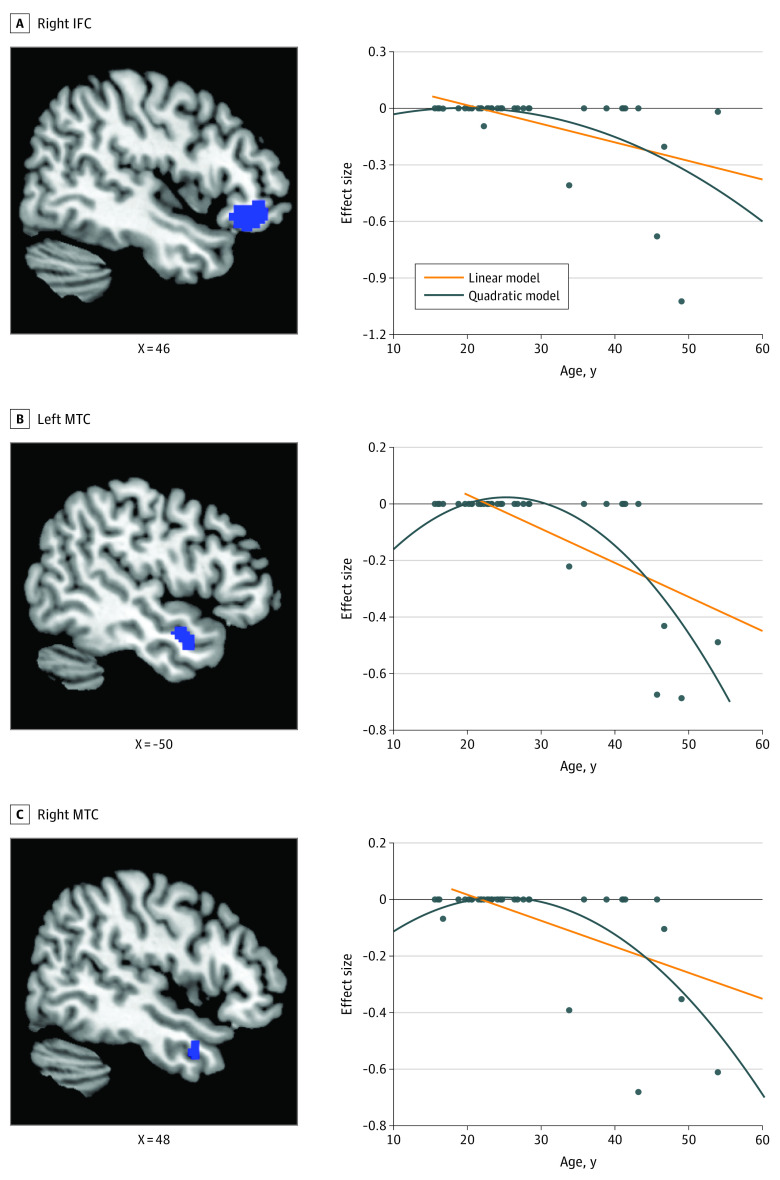
Meta-regression Results in the Combined Group Combining group including all studies regardless of stage of illness, showed an age-related pattern of cortical thickness reductions (relative to age-matched controls) in right pars orbitalis of inferior frontal cortex (IFC) (A), anterior left (B) and right lateral middle temporal cortex (MTC) (C). Both linear (orange line) and quadratic regression models (dark blue line) are shown. The quadratic model showed significantly better performance than the linear model in all regions.

Combining data sets across illness stages, reduced CTh in right pars orbitalis of inferior frontal cortex compared with healthy control individuals was correlated with older age (quadratic model: *R*^2^ = 0.28; *P* = .004) (Figure 4A). Cortical thickness reductions in individuals with left lateral MTC compared with healthy control individuals were correlated with greater age (linear model: *R*^2^ = 0.47; *P* < .001; quadratic model: *R*^2^ = 0.64; *P* < .001) (Figure 4B) and greater illness duration (linear model: *R*^2^ = 0.50; *P* < .001) (eFigure 7 in the Supplement). In the homologous right hemisphere, reduced CTh in right lateral MTC also was correlated with older age (linear model: *R*^2^ = 0.34; *P* < .001; quadratic model: *R*^2^ = 0.44; *P* < .001) (Figure 4C; eTable 6 in the Supplement). The quadratic model had a significantly lower root-mean-square error value than linear model in left lateral MTC (quadratic model: median [range], 0.12 [0.09-0.12]; linear model: median [range], 0.14 [0.12-0.14]; *z* = 5.37; *P* < .001) and right lateral MTC (quadratic model: median [range], 0.13 [0.09-0.13]; linear model: median [range], 0.14 [0.11-0.14]; *z* = 5.37; *P* < .001) (eTable 7 in the Supplement), indicating better performance of the quadratic model than the linear model in both regions. The findings for onset age or positive, negative, or general symptom ratings on CTh were not statistically significant in the combined group (eTable 6 in the Supplement).

### Jackknife, Heterogeneity, and Publication Bias Analysis

Whole-brain jackknife sensitivity analysis confirmed replicability and reliability of the findings (eResults 3, eTables 8-11 in the Supplement). Cortical thickness reductions in right insula showed high heterogeneity (*I*^2^ = 82.50%) in FEP groups and moderate heterogeneity (*I*^2^ = 45.00%) in the combined group, raising questions about the validity of findings in this region, so they were not considered further. The remaining regions with altered CTh showed low heterogeneity (*I*^2^ <15.00%) between studies (eResults in the Supplement). The Egger test of funnel plot asymmetry was not statistically significant when testing for publication bias (eResults, eFigures 8-11 in the Supplement).

## Discussion

Coordinate-based meta-analyses of CTh abnormalities across illness stages in SCZ identified robust CTh reductions in individuals with CHR, FEP, and long-term SCZ. There were no significant CTh differences between those with CHR and FEP. In contrast, alterations in CTh of individuals with long-term SCZ were more pronounced than seen in FEP studies in right inferior frontal cortex, left lateral temporal cortex (including superior temporal cortex and MTC), and right temporal pole. When combining studies regardless of illness stage, we observed accelerated age-related reductions of CTh in bilateral lateral MTC and right pars orbitalis compared with healthy control individuals. These findings did not demonstrate a robust reduction of CTh at illness onset but suggest a progressive age-related reduction in CTh later in the illness course.

### CTh Alterations at CHR and FEP

Individuals with CHR showed cortical thinning in bilateral medial prefrontal cortex, which plays an important role in cognitive functioning, emotion regulation, and motivation.^50^ Previous functional evidence suggested that hypoactivation of medial prefrontal cortex is related to anhedonia^51^ and poor episodic^52^ and working memory^53^ in SCZ, which are cognitive impairments already evident in individuals with CHR.^54,55^ Our findings were consistent with an earlier systematic review and meta-analysis that identified reduced medial prefrontal cortex volume in individuals with CHR.^56^ Moreover, greater gray matter loss in medial prefrontal cortex has been found in individuals with CHR who subsequently developed psychosis.^16^ Thus, our findings and previous volumetric studies converge in suggesting that anatomic alterations in medial prefrontal cortex precede the illness onset and thus may provide a marker of vulnerability to psychosis in individuals with CHR.^56,57^

Individuals with FEP demonstrated CTh reductions in right lateral superior temporal cortex and right anterior cingulate cortex relative to healthy control individuals. The superior temporal cortex contains primary and secondary auditory cortices and supports language comprehension.^58^ Decreased CTh,^59^ reduced gray matter volume,^60^ hypoactivation^61^ of superior temporal cortex, and reduced functional connectivity between superior temporal cortex and parietal areas^62^ have been associated with auditory verbal hallucinations, disordered thinking, and impaired speech recognition in SCZ. The anterior cingulate cortex is a major hub of the salience network engaged in cognitive, homeostatic, motivational, and affective functions.^63^ Gray matter loss^64^ and cortical thinning^65^ in this region have been linked to auditory hallucinations in FEP.

In no brain region did the severity of CTh alterations differ significantly between individuals with CHR and FEP. This lack of difference was seen when all individuals with CHR were considered—not just those who went on to manifest psychosis. This finding suggests that the CTh alterations seen in both groups for the most part reflect either a predisposing or neurodevelopmental feature already present by the time of prodrome manifestations. Furthermore, the lack of greater deficits in the FEP than CHR studies suggests that the onset of acute psychosis itself does not have robust neurotoxic outcomes associated with CTh.^66^

### CTh Reduction Associated With Long-term SCZ

Individuals with long-term SCZ exhibited cortical thinning in right inferior frontal cortex and bilateral temporal pole relative to healthy control individuals, in accordance with previous reports showing gray matter loss in these regions in individuals with long-term SCZ.^67^ The inferior frontal cortex is critical for affect modulation and empathy, which is impaired in SCZ.^68^ The temporal pole is part of the extended paralimbic system^69^ involved in emotion processing^70^ and interoceptive awareness.^71^ Gray matter volume reductions in paralimbic regions (including orbitofrontal cortex and temporal pole) have been related previously to cognitive and affective dysfunctions and clinical symptom severity in long-term SCZ.^72^

Individuals with long-term SCZ showed more pronounced CTh reductions in left lateral temporal cortex, right temporal pole, and right inferior frontal cortex than were seen in FEP studies considered relative to their own age-matched controls. These group differences suggest a progressive change in brain anatomy in SCZ relative to healthy control individuals over the years after illness onset. Such findings are of potential clinical importance, as greater gray matter volume and CTh reductions in frontotemporal regions have been associated with deteriorated cognitive deficits^73^ and poorer clinical outcomes^74^ that are a common and primary source of disability in this illness.^75^

Consistent with the possibility that differences between individuals with FEP and those with long-term SCZ may reflect aspects of disease progression, when pooling all included studies regardless of illness stage, we found accelerated age-related CTh reductions in bilateral lateral MTC and right pars orbitalis of inferior frontal cortex. These observations support the general idea of neuroprogression, but one localized to frontotemporal regions. Although the source of these progressive changes needs to be determined, because they may be directly related to illness pathophysiologic changes or be secondary to antipsychotic treatment effects or other environmental factors, the changes may themselves reflect a target for treatment development to reduce the longer-term morbidity associated with the disorder. The accelerated age-related CTh loss in frontotemporal cortex was often better modeled with a quadratic than linear model, suggesting accelerating progressive alterations in this region. Cortical thickness reductions of bilateral lateral MTC and right inferior frontal cortex appeared to accelerate in midlife, starting in the 40s, in accordance with previous volumetric studies showing steeper age-related gray matter volume loss in the frontotemporal regions.^76^

### Limitations

This study has limitations. First, although our meta-analytic approach provides the opportunity to combine cross-sectional studies across the literature to evaluate a potential regional pattern of progressive atrophic changes, longitudinal studies for confirmation may be warranted. Second, at first presentation of acute psychosis, differential diagnosis of affective and nonaffective disorders is challenging. Four of 12 FEP studies included patients with a current diagnosis of affective psychosis (with a proportion no more than 10%) (eTable 12 in the Supplement). This overlap in illness risk may also be relevant for CHR samples but could affect comparisons of FEP and long-term illness studies. Third, we were unable to explore medication therapy because precise information about dosage was not always available. Antipsychotic medications may contribute to the group differences and age outcomes in the long-term SCZ group. Fourth, the neuropathologic mechanism of cortical thinning has yet to be elucidated. Postmortem studies are needed to clarify the neural alterations leading to the findings observed in MRI studies. Such information could guide development of novel interventions to reduce these midlife brain changes in patients with SCZ. Fifth, our analyses focused on the thickness of cortical gray matter. Other anatomic features of neocortex merit attention in future studies, as does subcortical gray matter. Sixth, there is considerable heterogeneity in individuals meeting criteria for CHR that is yet to be resolved. Some will develop psychosis but most will not, and some will develop illnesses other than SCZ. Identifying discrete heterogeneity in individuals with CHR in association with brain features and clinical outcomes may ultimately lead to a better understanding of the similarities and differences of subgroups of patients with CHR compared with those who have FEP. We seem to be a few years from that point, as developing large samples and monitoring them longitudinally is an extensive effort, but examining discrete heterogeneity will greatly contribute to understanding of the CHR group. Seventh, despite documentation of limited interstudy heterogeneity in all regions but the insula, a small number of studies might influence regional study findings in our systematic review and meta-analysis. The variability across studies, although considered and evaluated statistically, raises caution in interpreting our systematic review and meta-analysis findings. This possibility will need to be evaluated as more published studies become available.

## Conclusions

The findings of this coordinate-based systematic review and meta-analysis identified CTh reductions in individuals with CHR that did not differ significantly from those observed in FEP studies. In addition, we observed CTh reductions in individuals with long-term SCZ compared with FEP that increased with age in frontotemporal regions and may reflect a neuroprogressive process. The lack of significant differences between individuals with CHR and FEP does not provide evidence indicating that first manifestation of acute psychosis is associated with robust CTh reductions. Together, these findings support understanding of the neural substrates of SCZ manifested at specific stages of illness and expand characterization of increasing illness-related neuroanatomic alterations over the illness course.

## Notes

Supplement. **eMethods.** Detailed Methods **eResults.** Detailed Results **eTable 1.** Overview of Published VBM and SBM Meta-analyses on Individuals at CHR, FEP, or Long-term SCZ **eTable 2.** The Checklist of Methodology Quality Assessment for the Included Studies **eTable 3.** Demographic and Clinical Characteristics of Studies Included in the Meta-analysis **eTable 4.** Results of the Meta-regression Analysis Within Each Group, Respectively **eTable 5.** Results of the Comparison Between CHR and Long-term SCZ Groups **eTable 6.** Results of the Meta-regression Analysis in the Combined Group **eTable 7.** Comparison of Mean Root-Mean-Square Error-Based Comparison Between Linear and Quadratic Models of Age Effects **eTable 8.** Results of the Jackknife Analysis in Studies for CHR Individuals **eTable 9.** Results of the Jackknife Analysis in Studies for FEP Individuals **eTable 10.** Results of the Jackknife Analysis in Studies for Long-term SCZ Individuals **eTable 11.** Results of the Jackknife Analysis in All Included Studies **eTable 12.** Proportion of Individuals With Affective Psychosis Included in the First-Episode Psychosis (FEP) Group **eFigure 1.** Differences in Cortical Thickness Between CHR Individuals and HCs **eFigure 2.** Differences in Cortical Thickness Between FEP Individuals and HCs **eFigure 3.** Differences in Cortical Thickness Between Long-term SCZ Individuals and HCs **eFigure 4.** Meta-regression Results in the Long-term SCZ Group **eFigure 5.** Differences in Cortical Thickness Between CHR and Long-term SCZ Groups **eFigure 6.** Differences in Cortical Thickness Between Combined Group and HCs **eFigure 7.** Meta-regression Results in the Combined Group **eFigure 8.** Results of Funnel Plot Analysis for the Meta-analysis of CHR Studies **eFigure 9.** Results of Funnel Plot Analysis for the Meta-analysis of FEP Studies **eFigure 10.** Results of Funnel Plot Analysis for the Meta-analysis of Long-term SCZ Studies **eFigure 11.** Results of Funnel Plot Analysis for the Pooled Meta-Analysis of All Included Studies
eReferences
