# Supplementary material for: Cortical Thickness Abnormalities at Different Stages of the Illness Course in Schizophrenia: A Systematic Review and Meta-analysis
Source: JAMA Psychiatry. 2022 Apr 27;79(6):560–70. doi: 10.1001/jamapsychiatry.2022.0799 (PMC9047772; doi:10.1001/jamapsychiatry.2022.0799)
Supplement: Supplement. — eMethods. Detailed Methods eResults. Detailed Results eTable 1. Overview of Published VBM and SBM Meta-analyses on Individuals at CHR, FEP, or Long-term SCZ eTable 2. The Checklist of Methodology Quality Assessment for the Included Studies eTable 3. Demographic and Clinical Characteristics of Studies Included in the Meta-analysis eTable 4. Results of the Meta-regression Analysis Within Each Group, Respectively eTable 5. Results of the Comparison Between CHR and Long-term SCZ Groups eTable 6. Results of the Meta-regression Analysis in the Combined Group eTable 7. Comparison of Mean Root-Mean-Square Error-Based Comparison Between Linear and Quadratic Models of Age Effects eTable 8. Results of the Jackknife Analysis in Studies for CHR Individuals eTable 9. Results of the Jackknife Analysis in Studies for FEP Individuals eTable 10. Results of the Jackknife Analysis in Studies for Long-term SCZ Individuals eTable 11. Results of the Jackknife Analysis in All Included Studies eTable 12. Proportion of Individuals With Affective Psychosis Included in the First-Episode Psychosis (FEP) Group eFigure 1. Differences in Cortical Thickness Between CHR Individuals and HCs eFigure 2. Differences in Cortical Thickness Between FEP Individuals and HCs eFigure 3. Differences in Cortical Thickness Between Long-term SCZ Individuals and HCs eFigure 4. Meta-regression Results in the Long-term SCZ Group eFigure 5. Differences in Cortical Thickness Between CHR and Long-term SCZ Groups eFigure 6. Differences in Cortical Thickness Between Combined Group and HCs eFigure 7. Meta-regression Results in the Combined Group eFigure 8. Results of Funnel Plot Analysis for the Meta-analysis of CHR Studies eFigure 9. Results of Funnel Plot Analysis for the Meta-analysis of FEP Studies eFigure 10. Results of Funnel Plot Analysis for the Meta-analysis of Long-term SCZ Studies eFigure 11. Results of Funnel Plot Analysis for the Pooled Meta-Analysis of All Included Studies eReferences [file jamapsychiatry-e220799-s001.pdf]

Supplementary Online Content

Zhao Y, Zhang Q, Shah C, et al. Cortical thickness abnormalities at different stages of the illness course in schizophrenia: a systematic review and meta-analysis. *JAMA Psychiatry*. Published online April 27, 2022. doi:10.1001/jamapsychiatry.2022.0799

eMethods. Detailed Methods
eResults. Detailed Results
eTable 1. Overview of Published VBM and SBM Meta-analyses on Individuals at CHR, FEP, or Long-term SCZ
eTable 2. The Checklist of Methodology Quality Assessment for the Included Studies
eTable 3. Demographic and Clinical Characteristics of Studies Included in the Meta-analysis
eTable 4. Results of the Meta-regression Analysis Within Each Group, Respectively
eTable 5. Results of the Comparison Between CHR and Long-term SCZ Groups
eTable 6. Results of the Meta-regression Analysis in the Combined Group
eTable 7. Comparison of Mean Root-Mean-Square Error-Based Comparison Between Linear and Quadratic Models of Age Effects
eTable 8. Results of the Jackknife Analysis in Studies for CHR Individuals
eTable 9. Results of the Jackknife Analysis in Studies for CHR Individuals
eTable 10. Results of the Jackknife Analysis in Studies for Long-term SCZ Individuals
eTable 11. Results of the Jackknife Analysis in All Included Studies
eTable 12. Proportion of Individuals With Affective Psychosis Included in the First-Episode Psychosis (FEP) Group
eFigure 1. Differences in Cortical Thickness Between CHR Individuals and HCs
eFigure 2. Differences in Cortical Thickness Between FEP Individuals and HCs
eFigure 3. Differences in Cortical Thickness Between Long-term SCZ Individuals and HCs
eFigure 4. Meta-regression Results in the Long-term SCZ Group
eFigure 5. Differences in Cortical Thickness Between CHR and Long-term SCZ Groups
eFigure 6. Differences in Cortical Thickness Between Combined Group and HCs
eFigure 7. Meta-regression Results in the Combined Group
eFigure 8. Results of Funnel Plot Analysis for the Meta-analysis of CHR Studies
eFigure 9. Results of Funnel Plot Analysis for the Meta-analysis of FEP Studies
eFigure 10. Results of Funnel Plot Analysis for the Meta-analysis of Long-term SCZ Studies
eFigure 11. Results of Funnel Plot Analysis for the Pooled Meta-Analysis of All Included Studies
eReferences

This supplementary material has been provided by the authors to give readers additional information about their work.

eMethods. Detailed Methods

eMethods 1. Search strategy and selection criteria

The meta-analysis was carried out complying with the Preferred Reporting Items for Systematic reviews and Meta-Analyses (PRISMA) guidelines (www.prisma-statement.org) (eTable 2) [1]. The protocol (registration number: CRD42021253875) was registered in the international prospective register of systematic reviews (www.crd.york.ac.uk/PROSPERO/). We conducted a systematic comprehensive literature search in PubMed, Embase, Web of Science, and Science Direct for studies on CTh published before June 15, 2021, using keywords such as "psychosis" or "psychotic" or "schizophrenia" or "schizoaffective" or "schizophreniform" plus "cortical thickness" or "thickness". Additional publications were identified by manual search in the reference lists. Two authors (Y.J.Z and Q.Z) independently conducted the literature search to ensure comprehensiveness. Any inconsistencies during the literature search were discussed and a consensus decision was reached about the appropriateness of the study for this meta-analysis.

Inclusion criteria were as follows: (a) studies published in English in a peer-reviewed journal; (b) included individuals with CHR for psychosis, or FEP, or long-term SCZ (which we defined studies where participants had illness duration greater than five years); (c) analyzed whole-brain CTh; and (d) provided the coordinates of significant clusters in Montreal Neurological Institute (MNI) or Talairach space. We excluded: (a) theoretical papers, case reports, reviews, and meta-analyses; (b) studies of individuals at genetic high risk for psychosis; (c) limited to region of interest analysis; (d) no statistical comparisons of CTh between individuals with CHR/FEP/long-term SCZ and HCs; and (e) peak coordinates of effects were not available even after contacting the authors. For longitudinal studies, only baseline data were included to avoid bias towards the effects of interventions or illness progression. For studies with multiple publications from overlapping samples, the one with the largest sample was included. For studies where multiple independent subgroups match criteria for one or more of the three illness stages of interest, the appropriate coordinates were included as separate datasets; if the coordinates of findings between subgroups and HCs were not available, we included the coordinates between the combined patient group (if they were within the range of one of our three illness stages) and HCs as one dataset. Eligible studies that reported no group differences were also included and estimated conservatively to have a null effect size in SDM.

eMethods 2. Quality assessment and data recording

A 12-parameter protocol was used to record average demographic and clinical characteristics of participants (sample size, gender, age, age of onset, illness duration, symptom severity, and medication status) and basic methodological information (statistical threshold of main findings and the method used to correct whole-brain results for multiple comparisons) (eTable 3). In the 12-point checklist [2], each point was scored 1 as fully met, 0.5 as partially met, or 0 as unfulfilled, respectively. Any study scoring >6.0 was included in the present meta-analysis. The checklist was not designed to critique the investigators or the work itself, but to provide an objective indication of the rigor of the individual studies [2]. We also extracted the coordinates of significant findings and statistical values related to effect size (e.g. t statistics, Z score, or P value) for SDM calculations [3].

eMethods 3. SDM method of meta-analysis

Meta-analyses of CTh abnormalities were conducted using SDM software (version 5.15). The details of the SDM method have been described elsewhere [4,5], but we summarize the approach here. First, SDM uses the coordinates of cluster peaks and the effect sizes of significant differences between participants and controls to create an effect-size signed map for each study utilizing an anisotropic Gaussian kernel. When selecting coordinates, the same threshold was used throughout the whole brain in each study to avoid bias towards regions with liberal thresholds. Eligible studies that reported no group differences were also included and estimated conservatively to have a null effect size in SDM. We used the "VBM (voxel-based morphometry) - gray matter" modality, "gray matter" correlation template, and "FreeSurfer" mask to increase the accuracy of effect size maps, which restricted maps to cortical gray matter [6]. Next, SDM was used to perform a random-effects analysis to obtain the mean map, combining data of each included study with both positive and negative differences included in the same map [4]. We used SDM's default thresholds (voxel threshold $P<0.005$ with peak $Z>1$ and a cluster extent of ten voxels) to display results in MNI coordinates.

eMethods 4. Jackknife, heterogeneity, and publication bias analysis

To test the replicability of results, whole-brain jackknife sensitivity analysis was conducted by repeating the main analysis N times (N=number of datasets in the meta-analysis), discarding one dataset at a time to determine whether the results remained significant [7]. Between-study heterogeneity was estimated using I^2 statistic. Publication bias was examined with Egger tests to assess the asymmetry of funnel plots for each significant cluster of patient-control comparisons, in which any result showing $P<0.05$ was judged to have significant publication bias [8].

eMethods 5. Meta-regression analysis

Meta-regression analyses were performed in the combined sample. In secondary exploratory studies, similar analyses were conducted in each group separately. The effect size values of each cluster were extracted from the SDM software and used for regression analyses with clinical and demographic variables. Clinical variables were not included in meta-regression analysis if data were available for fewer than nine studies [7], and thus only one variable (i.e., age) was explored in the CHR group and two variables (i.e., age and illness duration) were explored in FEP group and long-term SCZ group. Six variables (i.e., age, onset age, illness duration, positive, negative, and general scores of Positive and Negative Syndrome Scale [PANSS]) were explored in the combined group. Notably, since CHR group had no illness duration, meta-regression of this variable was performed only using data from the FEP and long-term SCZ groups. Aging effects on the human brain are believed to follow a nonlinear trajectory even through midlife in some brain regions [9], perhaps even more so in SCZ patients [10]. Thus, nonlinear (i.e., a quadratic model) regression models were further examined when testing for age relationships. Bonferroni adjustments corrected for the number of variables examined and the number of clusters. Therefore, the corrected P threshold for the CHR group, FEP group, long-term SCZ group, and the combined group was 0.05, 0.0083, 0.0063, and 0.0017 for linear regression analysis; and 0.05, 0.017, 0.0125, and 0.01 for nonlinear regression analysis of age, respectively. If both linear and quadratic models for age effects were significant for a cluster, performance of the two regression models was evaluated by their root-mean-square error (RMSE) (i.e., the standard deviation of the residual) with a leave-one-dataset-out cross-validation strategy, then RMSE values of the two models were compared using a paired Wilcoxon signed-rank test ($P<0.05$).

eResults. Detailed Results

eResults 1. Results of quality assessments

The mean score of CHR studies was 9.7, 9.33 for FEP studies, and 9.95 for long-term SCZ studies (eTable 3). Quality assessment items that deducted the most scores of these studies were lack of desired information about medication status, comorbidity, coordinates availability, acquisition parameters, and subtype status.

eResults 2. Characteristics of studies included in the meta-analysis

Age did not differ between patients and controls in each included study, nor between studies in each of our three separate meta-analyses or in the pooled meta-analysis (all P>0.05). For FEP and long-term SCZ groups, there were no significant differences in sex ratio between patients and HCs in each included study (all P>0.05), nor in FEP (P=0.1702) or long-term SCZ (P=0.1156) meta-analyses. For the CHR group, three original studies showed significant between-group differences in sex ratio [11-13], as did our meta-analysis of CHR studies (P<0.001). Three subdirectories of eTable 4 summarize the demographic and clinical characteristics of each included study for CHR, FEP, and long-term SCZ groups, respectively.

1. Characteristics of ten studies in CHR individuals

Among the ten included studies in CHR individuals, seven were cross-sectional and three were longitudinal. For these three longitudinal studies [14-16], only baseline data were included. Two of the ten studies subdivided CHR samples into subgroups based on whether they converted to frank psychosis [14,15] and had psychotic symptoms [17]. Notably, different diagnostic criteria were used to define the CHR participants. Five studies used the Structured Interview for Prodromal Syndromes (SIPS) criteria [14,15,17-19], three used the Comprehensive Assessment of At-Risk Mental States (CAARMS) criteria [16,20,21], one used Personal Assessment and Crisis Evaluation (PACE) criteria [11], and the remaining one used the Early Detection and Intervention in Psychosis (TIPS) [22].

CHR individuals of eight studies were medicated for the presentation of prodromal symptoms [11,14-17,19-21], of two were medication-naive [18,22]. Four of the seven cross-sectional studies identified CTh alterations in CHR individuals compared with HCs [11,19,20,22], while the other three cross-sectional studies did not find significant between-group differences [17,18,21], nor did the three longitudinal studies at baseline [14-16]. There were 13 datasets among the ten CHR studies included in the present meta-analysis.

2. Characteristics of 12 studies in individuals with FEP

Twelve studies were included in the FEP meta-analysis, in which eight were cross-sectional and four were longitudinally designed. Only baseline data of these four longitudinal studies were included [12,13,23,24]. FEP individuals of nine studies were medicated (less than a year) [11-13,24-29], of two were medication-naive [30,31], and one study recruited individuals with FEP who were at a wash-out period [23]. The illness duration of enrolled FEP individuals was specified in four studies [24,25,29,30] and unstated in two studies [11,13]. While other six studies described it as the duration of untreated psychosis since the treatment time was quite short (i.e., less than a year) [12,23,26-28,31]. Among the 12 FEP studies, three studies divided FEP individuals into subgroups based on drug class [25], cannabis use [24], and medication status [30], respectively. No significant case-control differences were reported in four of eight cross-sectional studies [26,28,30,31], nor in all four longitudinal studies at baseline [12,13,23,24]. Thirteen datasets were eventually included in the present FEP meta-analysis.

3. Characteristics of ten studies in individuals with long-term SCZ

Ten included studies on CTh of long-term SCZ comprised nine cross-sectional and one longitudinal study. Only baseline data of the longitudinal studies were included [32]. Eight studies enrolled all medicated individuals, among which six studies reported chlorpromazine equivalent dosages with a mean daily dose of 531.68 mg [33-38], one standardized the antipsychotic dosage using the defined daily dose with a mean unit of 1.53 [39], and the remaining one did not report the medication dosage [40]. One study recruited medication-naive individuals [10], and the remaining one used a mixture of antipsychotic-naive patients and some with previously treated but currently untreated individuals [32]. Three studies recruited two subgroups depending on the treatment response [33,39] and homogeneous subtype [36]. Significant CTh differences were reported between long-term SCZ individuals and HCs in all included studies except the longitudinal study at baseline [32]. There were 12 datasets among the ten studies included in the final long-term SCZ meta-analysis.

eResults 3. Results of jackknife, heterogeneity, and publication bias analysis

1. Results of the CHR group

In the CHR group, whole-brain jackknife sensitivity analysis showed that decreased CTh in bilateral mPFC was preserved in 11 of 13 combinations (eTable 9). This region showed very low heterogeneity (I^2=3.59%) between studies. Egger test of funnel plot asymmetry was not statistically significant (eFigure 8).

2. Results of the FEP group

In the FEP group, whole-brain jackknife sensitivity analysis showed that decreased CTh in the right lateral STC, ACC, and insula were preserved in 12, 11, and 13 of 13 combinations, respectively (eTable 10). The right lateral STC and ACC showed low heterogeneity (I^2=5.92%, 11.17%, respectively) between studies, while the right insula showed high heterogeneity (I^2=82.50%) between studies. Egger tests of funnel plot asymmetry were not statistically significant in the above three clusters (eFigure 9).

3. Results of the long-term SCZ group

In the long-term SCZ group, whole-brain jackknife sensitivity analysis showed that these CTh reductions were consistent, with effects in right insula and bilateral TP being preserved throughout all 12 combinations of datasets and those in the right pars orbitalis being significant in 11 combinations of 12 datasets (eTable 11). All findings showed low heterogeneity (I^2=3.79%, 3.82%, 5.33%, and 8.4% for right insula, right pars orbitalis, left temporal pole, and right temporal pole, respectively) between studies. Egger tests of funnel plot asymmetry were not statistically significant in any of the above four clusters (eFigure 10).

4. Results of the combined group

In the combined group with 38 datasets in total, whole-brain jackknife sensitivity analysis showed that decreased CTh in the right insula, left ACC, right pars orbitalis of IFC, left lateral MTC, and right lateral MTC were preserved in 38, 36, 36, 33, and 38 combinations of 38 datasets, respectively (eTable 12). The left ACC, right pars orbitalis, left lateral MTC, and right lateral MTC showed low heterogeneity (I^2=3.26%, 0.14%, 8.25%, and 15.16%, respectively) between studies, while the right insula showed moderate heterogeneity (I^2=45.00%) between studies. The Egger tests of funnel plot asymmetry were not statistically significant in the above five clusters (eFigure 11).

eTable 1. Overview of published VBM and SBM meta-analyses on individuals at CHR, FEP, or long-term SCZ

Stage	Study	methods	Sample size	Main results
CHR	Fortea et al. 2021 [41]	VBM & SBM	1248 CHR vs 1122 HCs; 153 CV vs 547 NCV	1. No significant differences in cortical GM were found in CHR individuals relative to HCs.<br>2. CV showed decreased cortical GM in right STC and MTC, ACC, and paracingulate gyrus than NCV.
	Liloia et al. 2021 [42]	VBM	580 CHR vs 6007 HCs	CHR individuals showed GMV reduction in right ACC than HCs.
	Hinney et al.2020 [43]	VBM	94 CV vs 513 NCV	No statistically significant differences in hippocampal volume between CV and NCV at baseline, but a trend of reduction of right hippocampus volume associated with the transition was found.
	Saunders et al. 2019 [44]	VBM	191 CHR vs 134 HCs	1. CHR individuals showed no significant differences in pituitary volume than HCs.<br>2. CV showed increased baseline pituitary volume than HCs.
	Smieskova et al. 2010 [45]	VBM	385 CHR vs 290 HCs	1. CV showed reduced regional GMV in the insula, ACC, PFC, and cerebellum than NCV.<br>2. CV showed larger global volumes than NCV.
FEP	Wen et al. 2021 [46]	SBM	624 FEP vs 505 HCs	FEP individuals showed CTh reductions in the right MTC extending to STC, insula, and ACC than HCs.
	Liloia et al. 2021 [42]	VBM	1636 FEP vs 6007 HCs	FEP individuals showed GMV reduction in the left precentral gyrus, left IFC, bilateral STC, bilateral transverse temporal gyrus, right MTC, bilateral insula, bilateral ACC, left parahippocampal gyrus, and left amygdala than HCs.
	Shah et al. 2017 [47]	VBM	801 FEP (449 AN-FEP and 352 AT-FEP) vs 957 HCs	1. AN-FEP individuals showed increased GM in the left inferior parietal gyri and left paracentral lobule, and decreased GM in the bilateral insula, right SFC, and left fusiform gyrus than HCs.<br>2. AT-FEP individuals showed increased GM in the right middle occipital gyrus, and right SFC and decreased GM in left ACC/paracingulate gyrus, left MTC, right postcentral gyrus, and left ITC than HCs.<br>3. GM in left supramarginal gyrus and left MTC were increased in AN-FEP but decreased in AT-FEP, whereas left MCC/paracingulate gyrus and right hippocampus GM was decreased in AN-FEP but increased in AT-FEP.
	Fraguas et al. 2014 [48]	VBM	156 FEP vs 163 HCs	FEP individuals showed progressive GMV changes in the frontal lobe than HCs.
	Olabi et al. 2012 [49]	VBM	555 FEP vs 621 HCs	FEP individuals showed GMV reductions in the bilateral caudate head, left insula, and bilateral uncus region.
	Vita et al. 2012 [50]	VBM	664 FEP vs 585 HCs	FEP individuals showed progressive GMV loss in the frontal, temporal and parietal lobes, and left Heschl gyrus than HCs.
	Chan et al. 2011 [51]	VBM	466 FEP vs 616 HCs	FEP individuals showed decreased GMV in the ACC and right insula than HCs.

	Leung et al. 2011 [52]	VBM	162 AN-FEP vs 165 HCs; 336 AT-FEP vs 484 HCs	1. AN-FEP showed GMV reductions in the bilateral caudate, insula, uncus, STC, and ITC; left PCC, precentral and SFC, and culmen; and right cingulate, middle frontal cortex, IFC, claustrum, and cerebellar tonsil than HCs.<br>2. AT-FEP showed GMV reductions in the bilateral insula, medial frontal cortex, IFC, and STC; left parahippocampal gyrus (amygdala), uncus, and ACC; and right thalamus, cingulate, precentral, and middle frontal gyrus than HCs.<br>3. AN-FEP individuals showed less GMV deficits in the bilateral insula, medial frontal, and IFC; left parahippocampal gyrus (amygdala) and STC; and right precentral gyrus, but more extensive GMV deficits in the bilateral caudate and ITC; left PCC, precentral, SFC, and culmen; right cingulate, middle frontal, and STC and claustrum than AT-FEP individuals.
	Ellison-Wright et al. 2008 [53]	VBM	224 FEP vs 248 HCs	FEP individuals showed decreased GMV in the thalamus, left uncus/amygdala region, the insula bilaterally, and the ACC than HCs.
**Long-term SCZ**	Liloia et al. 2021 [42]	VBM	2120 long-term SCZ vs 6007 HCs	Long-term SCZ individuals showed GMV reduction in right medial frontal cortex, left IFC, left STC, bilateral anterior insula, bilateral ACC, bilateral amygdala, head of left caudal nucleus, and medial dorsal nucleus of left thalamus than HCs.
	Chan et al. 2011 [51]	VBM	808 long-term SCZ vs 856 HCs	Long-term SCZ individuals showed decreased GMV in the ACC, right insula, right parahippocampus, left amygdala, left frontal lobe, left insula, thalamus, and left PCC than HCs.
	Ellison-Wright et al. 2008 [53]	VBM	1332 long-term SCZ vs 1293 HCs	Long-term SCZ individuals showed decreased GMV in the thalamus, left uncus/amygdala region, the insula bilaterally, and the ACC than HCs.

Abbreviations: CV, converters; NCV, nonconverters; CHR, clinical high-risk; SCZ, schizophrenia; VBM, voxel-based morphometry; GMV, gray matter volume; SBM, surface-based morphometry; CTh, cortical thickness; FEP, first-episode psychosis; AN-FEP, antipsychotic-naïve FEP; AT-FEP, antipsychotic-treated FEP; STC, superior temporal cortex; SFC, superior frontal cortex; ACC, anterior cingulate cortex; PFC, prefrontal cortex; IFC, inferior frontal cortex; PCC, posterior cingulate cortex; MTC, middle temporal cortex; ITC, inferior temporal cortex; MCC, middle cingulate cortex; vs, versus; HCs, healthy controls.

eTable 2. The checklist of methodology quality assessment for the included studies

1. The checklist of methodology quality assessment for the included studies in CHR individuals*

**12-point checklist** [2]		Bakker [18]	Buechler [17]	Cannon [14]	Dukart [11]	Gisselgård [22]	Jung [20]	Klauser [21]	Kwak [19]	Tognin [16]	Ziermans [15]
**Category 1: Subjects**											
1	Patients were evaluated prospectively, specific diagnostic criteria were applied, and demographic data were reported	1	1	1	1	1	1	1	1	1	1
2	Healthy comparison participants were evaluated prospectively; psychiatric and medical illnesses were excluded	1	1	1	1	1	1	1	1	1	1
3	Important variables (e.g., age, gender, drug status, illness duration, and symptom severity) were checked either via stratification or statistics	1	1	1	0.5	1	0.5	0.5	1	1	1
4	All patients were comorbidity free	0	0	0	0	1	0	0	0	1	0
5	All patients were medication naïve	1	0	0	0	1	0	0	0	0	0
6	Sample size per group: ≥ 20, scores 1; ≥ 10, scores 0.5	0.5	1	1	1	1	1	1	1	1	1
**Category 2: Methods for image acquisition and analysis**											
7	Magnet strength: 3T, scores 1; 1.5T, scores 0.5	1	1	1	1	0.5	0.5	1	1	0.5	0.5

8	The imaging technique used was clearly described so that it could be reproduced	1	1	1	1	1	1	1	1	1	1
9	Whole brain analysis was automated without a previously defined region	1	1	1	1	1	1	1	1	1	1
10	Spatial coordinates were reported in a standard space (e.g., Talairach or MNI coordinates)	0	1	0	1	1	1	1	1	1	0
**Category 3: Results and conclusions**											
11	Statistical results were corrected for multiple comparison scores 1, uncorrected scores 0.5	1	1	1	1	1	1	1	1	1	1
12	Conclusions were consistent with the results obtained, and the limitations were discussed	1	1	1	1	1	1	1	1	1	1
**Total score**		9.5	10.0	9.0	9.5	11.5	9.0	9.5	10.0	10.5	8.5

*Note: Each point was scored as 1, 0.5, or 0 if the criteria were fully met, partially met, or unfulfilled, respectively, and any study scoring >6.0 was included in the present meta-analysis.

2. The checklist of methodology quality assessment for the included studies in FEP individuals*.

**12-point checklist** [2]		Ansell [25]	Buchy [23]	Dukart [11]	Gutierrez-Galve [26]	Haukvik [12]	Lesh [30]	Lin [31]	Rais [24]	Reniers [13]	Scanlon [27]	Thormodsen [28]	Voets [29]
**Category 1: Subjects**													
1	Patients were evaluated prospectively, specific diagnostic criteria were applied, and demographic data were reported	1	1	1	1	1	1	1	1	1	1	1	1
2	Healthy comparison participants were evaluated prospectively; psychiatric and medical illnesses were excluded	1	1	1	1	1	1	1	1	1	1	1	1
3	Important variables (e.g., age, gender, drug status, illness duration, and symptom severity) were checked either via stratification or statistics	1	1	0.5	1	1	1	1	1	0.5	1	0.5	1
4	All patients were comorbidity free	0	0	0	0	1	0	0	0	1	0	0	0
5	All patients were medication naïve	1	1	0	0	0	1	1	0	0	0	0	0
6	Sample size per group: ≥ 20, scores 1; ≥ 10, scores 0.5	1	1	1	1	1	1	1	1	1	1	1	1
**Category 2: Methods for image acquisition and analysis**													

7	Magnet strength: 3T, scores 1; 1.5T, scores 0.5	0.5	0.5	1	0.5	0.5	0.5	1	0.5	1	0.5	0.5	0.5
8	The imaging technique used was clearly described so that it could be reproduced	1	1	1	1	1	1	1	1	1	1	1	1
9	Whole brain analysis was automated without a previously defined region	1	1	1	1	1	1	1	1	1	1	1	1
10	Spatial coordinates were reported in a standard space (e.g., Talairach or MNI coordinates)	1	0	1	0	0	0	0	0	0	0	1	1
**Category 3: Results and conclusions**													
11	Statistical results were corrected for multiple comparison scores 1, uncorrected scores 0.5	1	1	1	1	1	1	1	1	1	1	1	1
12	Conclusions were consistent with the results obtained, and the limitations were discussed	1	1	1	1	1	1	1	1	1	1	1	1
**Total score**		10.5	9.5	9.5	8.5	9.5	9.5	10	8.5	9.5	9.5	9	9.5

*Note: Each point was scored as 1, 0.5, or 0 if the criteria were fully met, partially met, or unfulfilled, respectively, and any study scoring >6.0 was included in the present meta-analysis.

3. The checklist of methodology quality assessment for the included studies in long-term SCZ individuals*.

12-point checklist [2]		Barry [33]	Green [40]	Kong [34]	Landin-Romero [38]	Madre [35]	Nelson [32]	Quide [37]	Xie [36]	Zhang [10]	Zugman [39]
**Category 1: Subjects**											
1	Patients were evaluated prospectively, specific diagnostic criteria were applied, and demographic data were reported	1	1	1	1	1	1	1	1	1	1
2	Healthy comparison participants were evaluated prospectively; psychiatric and medical illnesses were excluded	1	1	1	1	1	1	1	1	1	1
3	Important variables (e.g., age, gender, drug status, illness duration, and symptom severity) were checked either via stratification or statistics	1	1	0.5	0.5	1	1	1	1	1	0.5
4	All patients were comorbidity free	0	0	0	0	0	0	0	1	1	0
5	All patients were medication naïve	0	0	0	0	0	0	0	0	1	0
6	Sample size per group: ≥ 20, scores 1; ≥ 10, scores 0.5	1	1	1	1	1	1	1	1	1	1
**Category 2: Methods for image acquisition and analysis**											

7	Magnet strength: 3T, scores 1; 1.5T, scores 0.5	1	0.5	1	0.5	0.5	1	1	1	1	0.5
8	The imaging technique used was clearly described so that it could be reproduced	1	1	1	1	1	1	1	1	1	1
9	Whole brain analysis was automated without a previously defined region	1	1	1	1	1	1	1	1	1	1
10	Spatial coordinates were reported in a standard space (e.g., Talairach or MNI coordinates)	1	1	1	1	1	1	1	1	1	1
**Category 3: Results and conclusions**											
11	Statistical results were corrected for multiple comparison scores 1, uncorrected scores 0.5	1	1	1	1	1	1	1	1	1	1
12	Conclusions were consistent with the results obtained, and the limitations were discussed	1	1	1	1	1	1	1	1	1	1
**Total score**		10.0	9.5	9.5	9.0	9.5	10.0	10.0	11.0	12.0	9.0

*Note: Each point was scored as 1, 0.5, or 0 if the criteria were fully met, partially met, or unfulfilled, respectively, and any study scoring >6.0 was included in the present meta-analysis.

eTable 3. Demographic and clinical characteristics of studies included in the meta-analysis

1. Demographic and clinical characteristics of the ten studies of individuals with CHR features of psychosis

Study	Number (female)		Mean age, y		Symptom severity	Medication status (medicated number/CPZ equivalents (mg/day))	Statistical threshold (correction)
	CHR	HC	CHR	HC			
Bakker et al. 2016	18 (9)	24 (10)	22.7	23.4	PANSS: 68.2 (Total), 11.3 (Pos), 11.8 (Neg), 45.1 (Gen)	all naive	P < 0.05 (FDR)
Buechler et al. 2020 - NBS	39 (14)	34 (18)	21.79	21.76	SIPS: 38.24 (Total), 10.67 (Pos), 13.59 (Neg), 8.9 (Gen), 5.08 (Dis); GAF: 50.33	12/40.33	P < 0.05 (MCS)
Buechler et al. 2020 - BS	46 (23)	34 (18)	22.7	21.76	SIPS: 26.12 (Total), 4.67 (Pos), 11.24 (Neg), 7.28 (Gen), 2.93 (Dis); GAF: 57.98	8/21.66	P < 0.05 (MCS)
Cannon et al. 2015 - CV	35 (10)	135 (62)	18.8	20.5	SIPS: NA (Total), 13.5 (Pos), NA (Neg), NA (Gen), NA (Dis)	35/93.3	P < 0.05 (FDR)
Cannon et al. 2015 - NCV	239 (93)	135 (62)	19.7	20.5	SIPS: NA (Total), 11.9 (Pos), NA (Neg), NA (Gen), NA (Dis)	239/97.5	P < 0.05 (FDR)
Dukart et al. 2017	59 (16)	26 (14)	24.7	27.7	BPRS: 39.4; SANS: 11	23/NA	P < 0.05 (alpha-sim)
Gisselgård et al. 2018	41 (25)	37 (18)	16.7	16.9	SIPS: 33.1(Total), 10.1 (Pos), 10.9 (Neg), 8.8 (Gen), 3.3 (Dis); GAF: 49.9	all naive	P < 0.005 (MCS)
Jung et al. 2011	29 (14)	29 (14)	22.24	23.24	PANSS: 53.66 (Total), 12.72 (Pos), 11.72 (Neg), 29.17 (Gen); CAARMS: 37.41	10/NA	P < 0.01 (FDR)
Klauser et al. 2015	69 (22)	32 (15)	21.52	22.97	CAARMS: 16.33	37/NA	P < 0.05 (FDR)
Kwak et al. 2019	74 (20)	34 (14)	20.61	20.29	SOPS: 10.03 (Pos), 14.31 (Neg), 7.18 (Gen), 4.38(Dis)	28/NA	P < 0.05 (MCS)
Tognin et al. 2014 - CV	50 (13)	150 (51)	22.9	23.4	PANSS: 57.25 (Total), 12.06 (Pos), 15.44 (Neg), 29.75 (Gen); CAARMS: 8.37; GAF: 46.5; BPRS: 33.08; SANS: 21.26	5/NA	P < 0.05 (FWE)
Tognin et al. 2014 - NCV	117 (49)	150 (51)	23.3	23.4	PANSS: 49.25 (Total), 10.27 (Pos), 12.02 (Neg), 26.95 (Gen); CAARMS: 6.82; GAF: 58.12; BPRS: 31.3; SANS: 19.48	21/NA	P < 0.05 (FWE)
Ziermans et al. 2012	43 (14)	30 (15)	15.6	15.9	GAF: 59; BSABS: 20.5	21/NA	P < 0.05 (FDR)

Abbreviations: BPRS, Brief Psychiatric Rating Scale; BS, basic symptoms; BSABS, Bonn Scale for the Assessment of Basic Symptoms; CAARMS, Comprehensive Assessment of At-Risk Mental States; CHR, clinical high-risk; CPZ, chlorpromazine; CV, converters; Dis, disorganization; FDR, False Discovery Rate; FWE, Family-Wise Error; GAF, global assessment of functioning; Gen, general; HCs, healthy controls; MCS, Monte Carlo simulation; NA, not available; NBS, not only basic symptoms; NCV, non-converters; Neg, negative; PANSS, Positive and

Negative Syndrome Scale; Pos, positive; SANS, Scale for Assessment of Negative Symptoms; SIPS, Structured interview for prodromal syndromes; SOPS, Scale of Prodromal Symptoms criteria.

2. Demographic and clinical characteristics of the 12 studies of individuals with FEP.

Study	Number (female)			Mean age, y		Age of onset, y	Illness duration, y	PANSS scores				Medication status (medicated number/CPZ equivalents (mg/day))	Statistical threshold (correction)
	FEP	HC		FEP	HC			Total	Pos	Neg	Gen		
Ansell et al. 2015 - FGA	25 (8)	28 (11)		21.93	21.07	21.6	0.12	86	22.4	22	41.6	25/160.96	P < 0.05 (FWE)
Ansell et al. 2015 - SGA	27 (9)	28 (11)		21.95	21.07	21.4	0.25	88.6	22.4	21.9	44.3	27/251.85	P < 0.05 (FWE)
Buchy et al. 2017	130 (37)	52 (15)		24.1	24.3	NA	0.93	NA	NA	NA	NA	wash-out	P < 0.005 (RFT)
Dukart et al. 2017	59 (17)	26 (14)		26.4	27.7	NA	NA	NA	NA	NA	NA	40/216	P < 0.05 (alpha-sim)
Gutierrez-Galve et al. 2010	37 (12)	38 (16)		26.8	25	NA	0.87	NA	NA	NA	NA	37/NA	P < 0.05 (FDR)
Haukvik et al. 2016	79 (27)	82 (28)		27.6	29.3	23.8	2.36	60.6	14.9	14	31.7	79/NA	P < 0.05 (FDR)
Lesh et al. 2015	22 (3)	37 (9)		20.2	19.7	NA	0.58	NA	NA	NA	NA	all naive	P < 0.05 (MCS)
Lin et al. 2019	145 (76)	147 (76)		24.5	25.9	23.6	0.9	93.2	25.2	19.9	48.1	all naive	P < 0.05 (FDR)
Rais et al. 2010	32 (6)	31 (6)		23.28	24.72	21.54	1.07	NA	17.23	18.18	NA	51/NA	P < 0.1 (FDR)
Reniers et al. 2014	22 (4)	22 (4)		20.64	22.48	NA	NA	NA	NA	NA	NA	14/200	P < 0.002 (MCS)
Scanlon et al. 2014	46 (14)	46 (13)		28.4	28.6	NA	1.17	65	17	15	33	46/224	P < 0.05 (FDR)
Thormodsen et al. 2013	22 (12)	32 (17)		16.2	15.9	NA	0.41	57	14.4	12.7	29.9	17/1.47*	P < 0.05 (FDR)
Voets et al. 2008	25 (7)	25 (8)		16	16	15	1.4	NA	NA	NA	NA	25/340	P < 0.05 (FDR)

*Defined Daily Dosages of antipsychotic medication use were calculated using the guidelines from the WHO (https://www.whocc.no/atc_ddd_index/).

Abbreviations: CPZ, chlorpromazine; FDR, False Discovery Rate; FEP, first-episode psychosis; FGA, first-generation antipsychotic; FWE, Family-Wise Error; Gen, general; HC, healthy controls; MCS, Monte Carlo simulation; NA, not available; Neg, negative; PANSS, Positive and Negative Syndrome Scale; Pos, positive; RFT, random field theory; SGA, second-generation antipsychotic.

3. Demographic and clinical characteristics of the ten studies of individuals with long-term SCZ.

Study	Number (female)		Mean age, y		Age of onset, y	Illness duration, y	PANSS scores				Medication status (medicated number/CPZ equivalents (mg/day))	Statistical threshold (correction)
	Long-term SCZ	HC	Long-term SCZ	HC			Total	Pos	Neg	Gen		
Barry et al. 2019	42 (6)	23 (6)	41.4	38.4	26.85	14.8	61.55	15.6	16.3	29.3	42/331.9	P < 0.05 (RFT)
Green et al. 2016	22 (12)	22 (12)	38.86	39.57	22.89	13.78	73.9	18.29	17.43	38.19	22/NA	P < 0.07 (MCS)
Kong et al. 2015	22 (6)	20 (8)	53.95	52.75	NA	31.54	NA	NA	NA	NA	22/542.53	P < 0.05 (FDR)
Landin-Romero et al. 2017	44 (18)	45 (19)	43.18	43.02	NA	20.18	32.86	14.2	11.66	7	44/709.01	P < 0.06 (CWC)
Madre et al. 2020	128 (54)	127 (54)	41	39	22	18	NA	NA	NA	NA	128/585	P < 0.05 (CWC)
Nelson et al. 2020	34 (9)	23 (4)	28.32	27.48	22.08	15	NA	NA	NA	NA	22 naive, 12 wash out	P < 0.05 (MCS)
Quide et al. 2019	60 (24)	61 (27)	41.16	35.98	22.53	18.33	56.35	14.05	14.78	27.52	125 /557.77	P < 0.05 (FWE)
Xie et al. 2019 - DS	33 (0)	41 (0)	49.03	45.78	22.03	27	NA	NA	NA	NA	33/467.73	P < 0.05 (FDR)
Xie et al. 2019 - NDS	41 (0)	41 (0)	45.71	45.78	22.39	23.32	NA	NA	NA	NA	41/527.8	P < 0.05 (FDR)
Zhang et al. 2015	25 (11)	33 (15)	46.68	46.21	25.64	21.04	88.71	24.08	22.5	41.54	all naive	P < 0.05 (MCS)
Zugman et al. 2013 - NTR	67 (22)	80 (27)	35.81	33.46	NA	12.21	54.58	11.61	15.39	27.73	67/1.34*	P < 0.05 (MCS)
Zugman et al. 2013 - TR	61 (21)	80 (27)	33.8	33.46	NA	12.9	63.41	14.1	18.67	30.39	61/1.71*	P < 0.05 (MCS)

*Defined Daily Dosages of antipsychotic medication use were calculated using the guidelines from the WHO (https://www.whocc.no/atc_ddd_index/).

Abbreviations: CPZ, chlorpromazine; CWC, cluster-wise correction; FDR, False Discovery Rate; FWE, Family-Wise Error; Gen, general; HC, healthy control; SCZ, schizophrenia; MCS, Monte Carlo simulation; NA, not available; NDS, nondeficit schizophrenia; Neg, negative; NTR, treatment response; PANSS, Positive and Negative Syndrome Scale; Pos, positive; RFT, random field theory; TR, treatment resistance.

eTable 4. Results of the meta-regression analysis within each group, respectively

Clusters		Variables		Linear model			Quadratic model	
				R^2	P		R^2	P
**CHR**								
	Left medial prefrontal cortex	age		0.003	0.86		0.11	0.56
**FEP**								
	Right lateral superior temporal cortex	age		0.001	0.93		0.43	0.06
	Right anterior cingulate cortex			0.17	0.16		0.17	0.39
	Right insula			0.01	0.75		0.09	0.64
	Right lateral superior temporal cortex	illness duration		0.09	0.38		-	-
	Right anterior cingulate cortex			0.02	0.69		-	-
	Right insula			0.16	0.31		-	-
**Long-term SCZ**								
	Right insula	age		0.07	0.41		0.07	0.72
	Right inferior frontal cortex, orbital part			0.22	0.13		0.22	0.33
	Right temporal pole, superior temporal cortex			0.37	0.04		0.44	0.07
	Left temporal pole, middle temporal cortex			0.43	0.02		0.59	0.02
	Right insula	**illness duration**		0.04	0.52		-	-
	Right inferior frontal cortex, orbital part			0.26	0.09		-	-
	Right temporal pole, superior temporal cortex			0.49	0.01		-	-
	**Left temporal pole, middle temporal cortex**			0.60	**0.003**		-	-

Note: A region that survived Bonferroni correction for multiple comparisons ($P<0.05$) is shown in bold font. The corrected P threshold for the CHR group, FEP group, and long-term SCZ group was 0.05, 0.0083, and 0.0063 for linear regression analysis; and 0.05, 0.017, and 0.0125 for nonlinear regression analysis of age, respectively.

Abbreviations: CHR, clinical high-risk; FEP, first-episode psychosis; SCZ, schizophrenia.

eTable 5. Results of the comparison between CHR and long-term SCZ groups

Region	MNI coordinate			SDM	*P*, uncorrected	Voxels	Cluster breakdown (voxels)
	x	y	z	*Z* score			
Long term-SCZ < CHR							
Right insula	40	4	2	-2.95	<0.001	1222	Right insula (714)
							Right rolandic operculum (190)
							Right inferior frontal gyrus, opercular part (148)
							Right lenticular nucleus, putamen (121)
							Right temporal pole, superior temporal gyrus (49)
Right inferior frontal cortex, orbital part	48	30	-12	-2.41	<0.001	368	Left inferior frontal cortex, orbital part (350)
							Left inferior frontal cortex, triangular part (18)
Left middle temporal cortex	-50	0	-20	-2.02	0.002	344	Left middle temporal cortex (231)
							Left superior temporal cortex (82)
							Left inferior temporal cortex (31)
Right temporal pole, middle temporal cortex	50	6	-32	-1.92	0.002	143	Right temporal pole, middle temporal cortex (108)
							Right inferior temporal cortex (26)
							Right temporal pole, superior temporal cortex (9)
Abbreviations: CHR, clinical high-risk; SCZ, schizophrenia; MNI, Montreal Neurological Institute.							

eTable 6. Results of the meta-regression analysis in the combined group

Clusters	Variables	Linear model R^2	Linear model P	Quadratic model R^2	Quadratic model P
Right insula	**age**	0.07	0.12	0.16	0.05
Left anterior cingulate cortex		0.02	0.42	0.03	0.64
**Right inferior frontal cortex, orbital part**		0.25	0.002	0.28	**0.004**
**Left lateral middle temporal cortex**		0.47	**<0.001**	0.64	**<0.001**
**Right lateral middle temporal cortex**		0.34	**<0.001**	0.44	**<0.001**
Right insula	onset age	0.07	0.38	-	-
Left anterior cingulate cortex		0.51	0.004	-	-
Right inferior frontal cortex, orbital part		0.001	0.92	-	-
Left lateral middle temporal cortex		0.02	0.66	-	-
Right lateral middle temporal cortex		0.004	0.83	-	-
Right insula	**illness duration**	0.05	0.33	-	-
Left anterior cingulate cortex		0.04	0.37	-	-
Right inferior frontal cortex, orbital part		0.26	0.01	-	-
**Left lateral middle temporal cortex**		0.50	**<0.001**	-	-
Right lateral middle temporal cortex		0.36	0.002	-	-
Right insula	positive symptom scores	0.07	0.33	-	-
Left anterior cingulate cortex		0.005	0.80	-	-
Right inferior frontal cortex, orbital part		0.001	0.89	-	-
Left lateral middle temporal cortex		0.09	0.27	-	-
Right lateral middle temporal cortex		0.000	0.98	-	-
Right insula	negative symptom scores	0.18	0.09	-	-
Left anterior cingulate cortex		0.001	0.92	-	-
Right inferior frontal cortex, orbital part		0.07	0.30	-	-
Left lateral middle temporal cortex		0.19	0.08	-	-
Right lateral middle temporal cortex		0.01	0.68	-	-
Right insula	general symptom scores	0.03	0.56	-	-
Left anterior cingulate cortex		0.000	1.00	-	-
Right inferior frontal cortex, orbital part		0.02	0.65	-	-
Left lateral middle temporal cortex		0.02	0.68	-	-
Right lateral middle temporal cortex		0.02	0.68	-	-

Note: Regions that survived Bonferroni correction for multiple comparisons ($P<0.05$) are shown in bold font. The corrected P threshold for the combined group was 0.0017 for linear regression analysis; and 0.01 for nonlinear regression analysis of age, respectively.

eTable 7. Comparison of mean root-mean-square error-based comparison between linear and quadratic models of age effects

Clusters		Variables		Linear model			Quadratic model			paired Wilcoxon signed-rank test	
				median	Range		median	Range		Z value	P
L MTC		Age		0.14	0.12-0.14		0.12	0.09-0.12		5.37	<0.001
R MTC		Age		0.14	0.11-0.14		0.13	0.09-0.13		5.37	<0.001

Abbreviations: L, left; MTC, middle temporal cortex; R, right

eTable 8. Results of the jackknife analysis in studies for CHR individuals

Discarded study	Decreased cortical thickness
	Bilateral medial prefrontal cortex
Bakker et al. 2016	Yes
Buechler et al. 2020 - BS	Yes
Buechler et al. 2020 - NBS	Yes
Cannon et al. 2015 - CV	Yes
Cannon et al. 2015 - NCV	Yes
Dukart et al. 2017	Yes
Gisselgård et al. 2018	Yes
Jung et al. 2011	No
Klauser et al. 2015	Yes
Kwak et al. 2019	No
Tognin et al. 2014 - CV	Yes
Tognin et al. 2014 - NCV	Yes
Ziermans et al. 2012	Yes
	11/13

Abbreviations: BS, basic symptoms; CHR, clinical high-risk; CV, converters; NBS, not only basic symptoms; NCV, non-converters.

eTable 9. Results of the jackknife analysis in studies for FEP individuals

Discarded study	Decreased cortical thickness		
	Right lateral superior temporal cortex	Right anterior cingulate cortex	Right insula
Ansell et al. 2015 - FGA	Yes	Yes	Yes
Ansell et al. 2015 - SGA	Yes	No	Yes
Buchy et al. 2017	Yes	Yes	Yes
Dukart et al. 2017	Yes	Yes	Yes
Gutierrez-Galve et al. 2010	Yes	Yes	Yes
Haukvik et al. 2016	Yes	Yes	Yes
Lesh et al. 2015	Yes	Yes	Yes
Lin et al. 2019	Yes	Yes	Yes
Rais et al. 2010	Yes	Yes	Yes
Reniers et al. 2014	Yes	Yes	Yes
Scanlon et al. 2014	No	Yes	Yes
Thormodsen et al. 2013	Yes	Yes	Yes
Voets et al. 2008	Yes	No	Yes
	12/13	11/13	13/13

Abbreviations: FEP, first-episode psychosis; FGA, first-generation antipsychotic; SGA, second-generation antipsychotic

eTable 10. Results of the jackknife analysis in studies for long-term SCZ individuals

Discarded study	Decreased cortical thickness			
	Right insula	Right pars orbitalis of inferior frontal cortex	Left temporal pole, middle temporal cortex	Right temporal pole, superior temporal cortex
Barry et al. 2019	Yes	Yes	Yes	Yes
Green et al. 2016	Yes	Yes	Yes	Yes
Kong et al. 2015	Yes	Yes	Yes	Yes
Madre et al. 2020	Yes	Yes	Yes	Yes
Nelson et al. 2020	Yes	Yes	Yes	Yes
Quide et al. 2019	Yes	Yes	Yes	Yes
Landin-Romero et al. 2017	Yes	Yes	Yes	Yes
Xie et al. 2019-DS	Yes	Yes	Yes	Yes
Xie et al. 2019-NDS	Yes	No	Yes	Yes
Zhang et al. 2015	Yes	Yes	Yes	Yes
Zugman et al. 2013-NTR	Yes	Yes	Yes	Yes
Zugman et al. 2013-TR	Yes	Yes	Yes	Yes
	12/12	11/12	12/12	12/12

Abbreviations: DS, deficit schizophrenia; SCZ, schizophrenia; NDS, nondeficit schizophrenia; NTR, treatment response; TR, treatment resistance.

eTable 11. Results of the jackknife analysis in all included studies

Discarded study	Decreased cortical thickness				
	Right insula	Left anterior cingulate cortex	Right pars orbitalis of inferior frontal cortex	Left lateral middle temporal cortex	Right lateral middle temporal corte x
Ansell et al. 2015 - FGA	Yes	Yes	Yes	Yes	Yes
Ansell et al. 2015 - SGA	Yes	Yes	Yes	Yes	Yes
Bakker et al. 2016	Yes	Yes	Yes	Yes	Yes
Barry et al. 2019	Yes	Yes	Yes	Yes	Yes
Buchy et al. 2017	Yes	Yes	Yes	Yes	Yes
Buechler et al. 2020 - BS	Yes	Yes	Yes	Yes	Yes
Buechler et al. 2020 - NBS	Yes	Yes	Yes	Yes	Yes
Cannon et al. 2015 - CV	Yes	Yes	Yes	Yes	Yes
Cannon et al. 2015 - NCV	Yes	Yes	Yes	Yes	Yes
Dukart et al. 2017 - FEP	Yes	Yes	Yes	Yes	Yes
Dukart et al. 2017 - CHR	Yes	Yes	Yes	Yes	Yes
Gisselgård et al. 2018	Yes	Yes	Yes	Yes	Yes
Green et al. 2016	Yes	Yes	Yes	Yes	Yes
Gutierrez-Galve et al. 2010	Yes	Yes	Yes	Yes	Yes
Haukvik et al. 2016	Yes	Yes	Yes	Yes	Yes
Jung et al. 2011 - CHR	Yes	Yes	Yes	Yes	Yes
Klauser et al. 2015	Yes	Yes	Yes	Yes	Yes
Kong et al. 2015	Yes	Yes	Yes	No	Yes
Kwak et al. 2019	Yes	Yes	Yes	Yes	Yes
Landin-Romero et al. 2017	Yes	Yes	Yes	Yes	Yes
Lesh et al. 2015	Yes	Yes	Yes	Yes	Yes
Lin et al. 2019	Yes	Yes	Yes	Yes	Yes
Madre et al. 2020	Yes	Yes	Yes	Yes	Yes
Nelson et al. 2020	Yes	Yes	Yes	Yes	Yes
Quide et al. 2019	Yes	Yes	Yes	Yes	Yes
Rais et al. 2010	Yes	Yes	Yes	Yes	Yes
Reniers et al. 2014	Yes	Yes	Yes	Yes	Yes
Scanlon et al. 2014	Yes	Yes	Yes	Yes	Yes
Thormodsen et al. 2013	Yes	Yes	Yes	Yes	Yes
Tognin et al. 2014 - CV	Yes	Yes	Yes	Yes	Yes
Tognin et al. 2014 - NCV	Yes	Yes	Yes	Yes	Yes
Voets et al. 2008	Yes	Yes	Yes	Yes	Yes
Xie et al. 2019 - DS	Yes	Yes	No	No	Yes
Xie et al. 2019 - NDS	Yes	Yes	No	No	Yes
Zhang et al. 2015	Yes	Yes	Yes	No	Yes
Ziermans et al. 2012	Yes	Yes	Yes	Yes	Yes
Zugman et al. 2013 - NTR	Yes	No	Yes	Yes	Yes
Zugman et al. 2013 - TR	Yes	No	Yes	No	Yes
	38/38	36/38	36/38	33/38	38/38

Abbreviations: CHR, clinical high-risk; FEP, first-episode psychosis; SGA, second-generation antipsychotic; NCV, non-converters;

BS, basic symptoms; CV, converters; DS, deficit schizophrenia; FGA, first-generation antipsychotic; NBS, not only basic symptoms; NDS, nondeficit schizophrenia; NTR, treatment response; SCZ, schizophrenia; TR, treatment resistance.

eTable 12. Proportion of individuals with affective psychosis included in the first-episode psychosis (FEP) group

Studies	Included samples of affective psychosis (number)	Proportion of affective psychosis in all FEP individuals
Buchy et al. 2017	Bipolar I with psychotic features (14)<br>Bipolar II with psychotic features (1)<br>Major depression with psychotic features (8)	65/671=9.70%
Haukvik et al. 2016	Bipolar I disorder with psychotic features (15)<br>Bipolar II disorder with psychotic features (2)<br>Bipolar unspecified (1)<br>Major depressive disorder with psychotic features (5)	
Reniers et al. 2014	Major depressive disorder with psychotic features (2)<br>Bipolar unspecified (1)<br>Bipolar disorder with psychotic features (1)	
Scanlon et al. 2014	Bipolar I disorder with psychotic features (9)<br>Major depressive disorder with psychotic features (6)	

eFigure 1. Differences in cortical thickness between CHR individuals and HCs

CTh reductions (cool color) in individuals with clinical high-risk (CHR) compared to healthy controls were found in the bilateral medial prefrontal cortex (mPFC). Color bar shows the SDM Z values. Abbreviations: L, left; R, right.

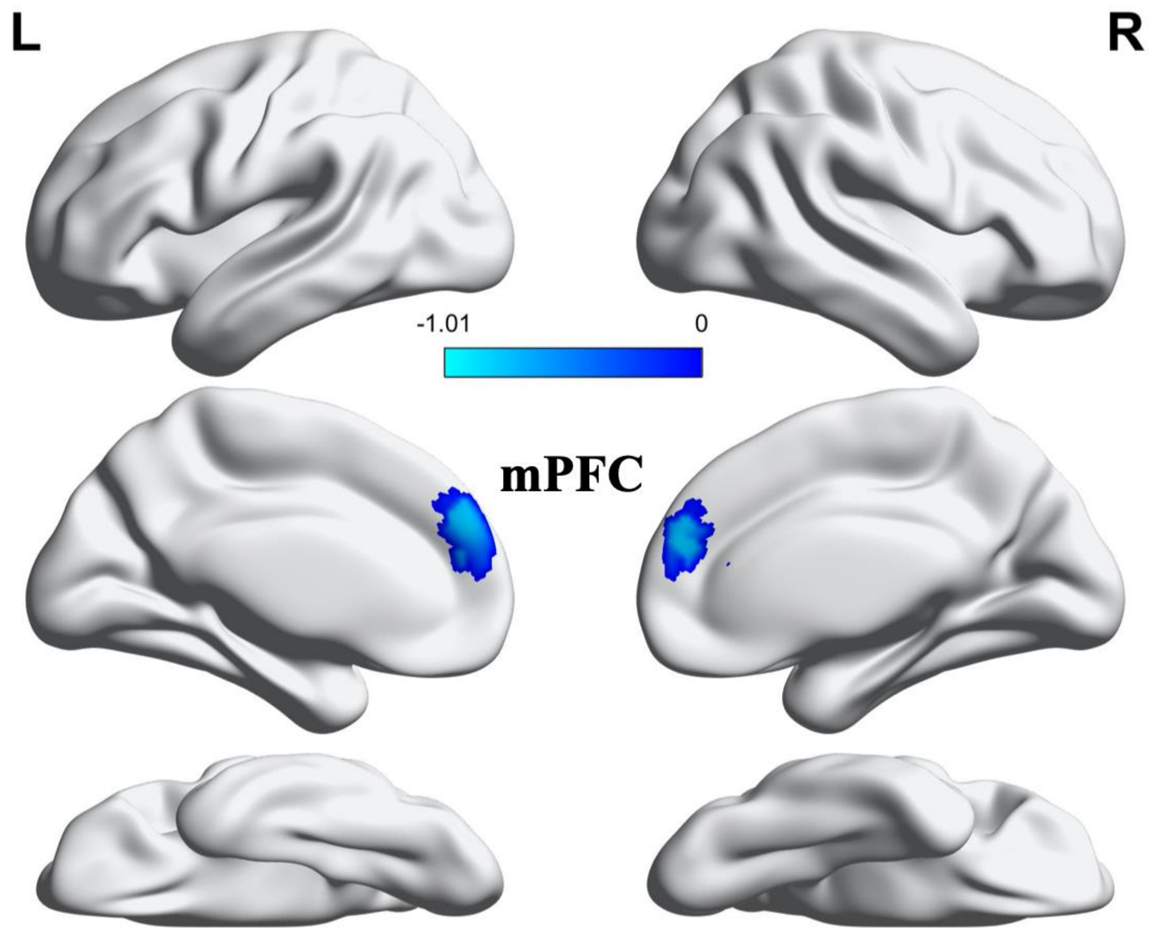

eFigure 2. Differences in cortical thickness between FEP individuals and HCs
CTh reductions (cool color) in individuals with first-episode psychosis (FEP) compared to healthy controls were found in the right lateral superior temporal cortex (STC), right anterior cingulate cortex (ACC), and right insula. Color bar shows the SDM Z values. Abbreviations: L, left; R, right.

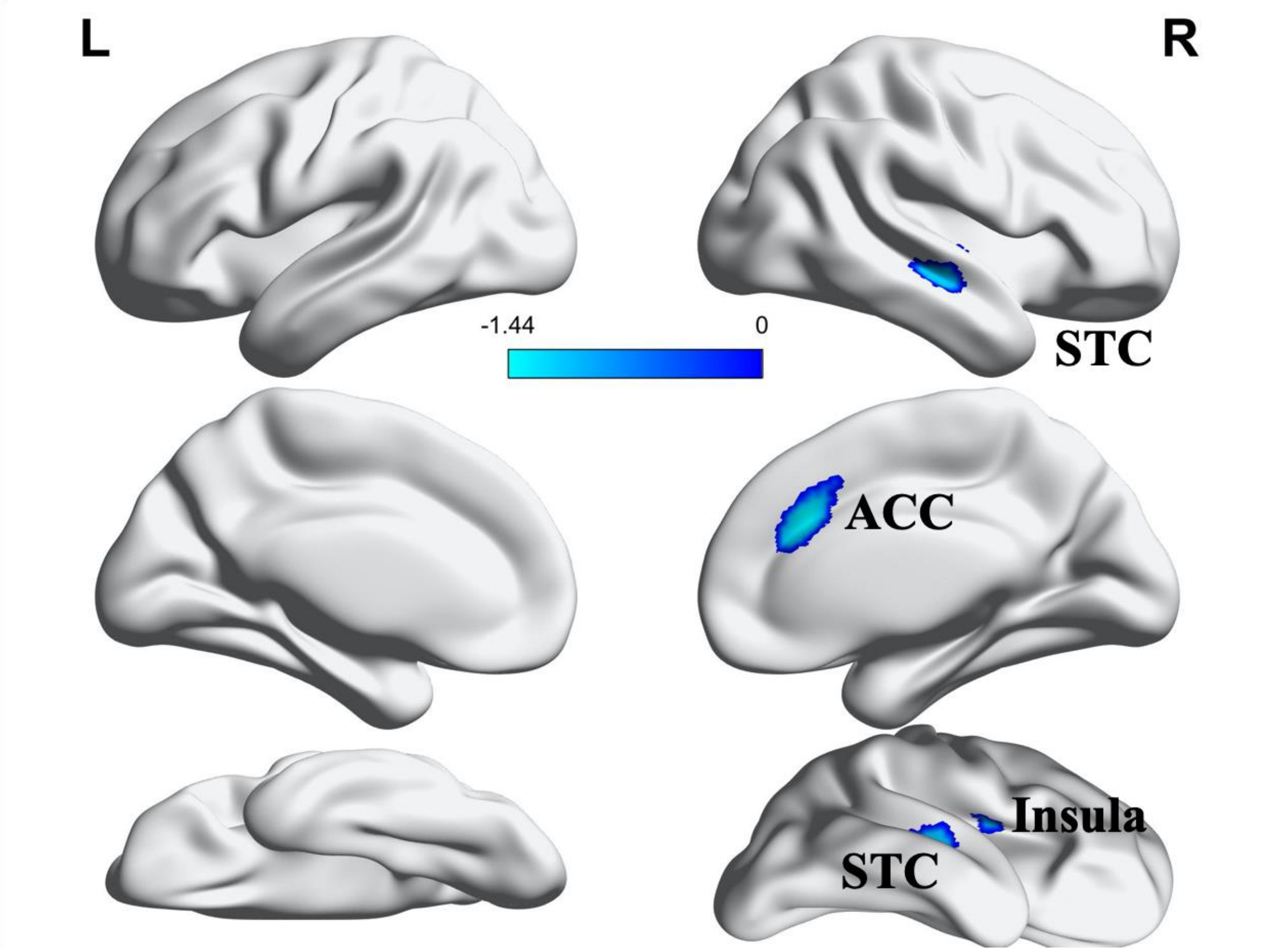

eFigure 3. Differences in cortical thickness between long-term SCZ individuals and HCs

CTh reductions (cool color) in individuals with long-term schizophrenia (SCZ) compared to healthy controls were found in the right insula extending to frontal operculum, right pars orbitalis of inferior frontal cortex (IFC), bilateral temporal pole (TP) (including anterior part of middle temporal cortex, superior temporal cortex, and inferior temporal cortex). Color bar shows the SDM Z values. Abbreviations: L, left; R, right.

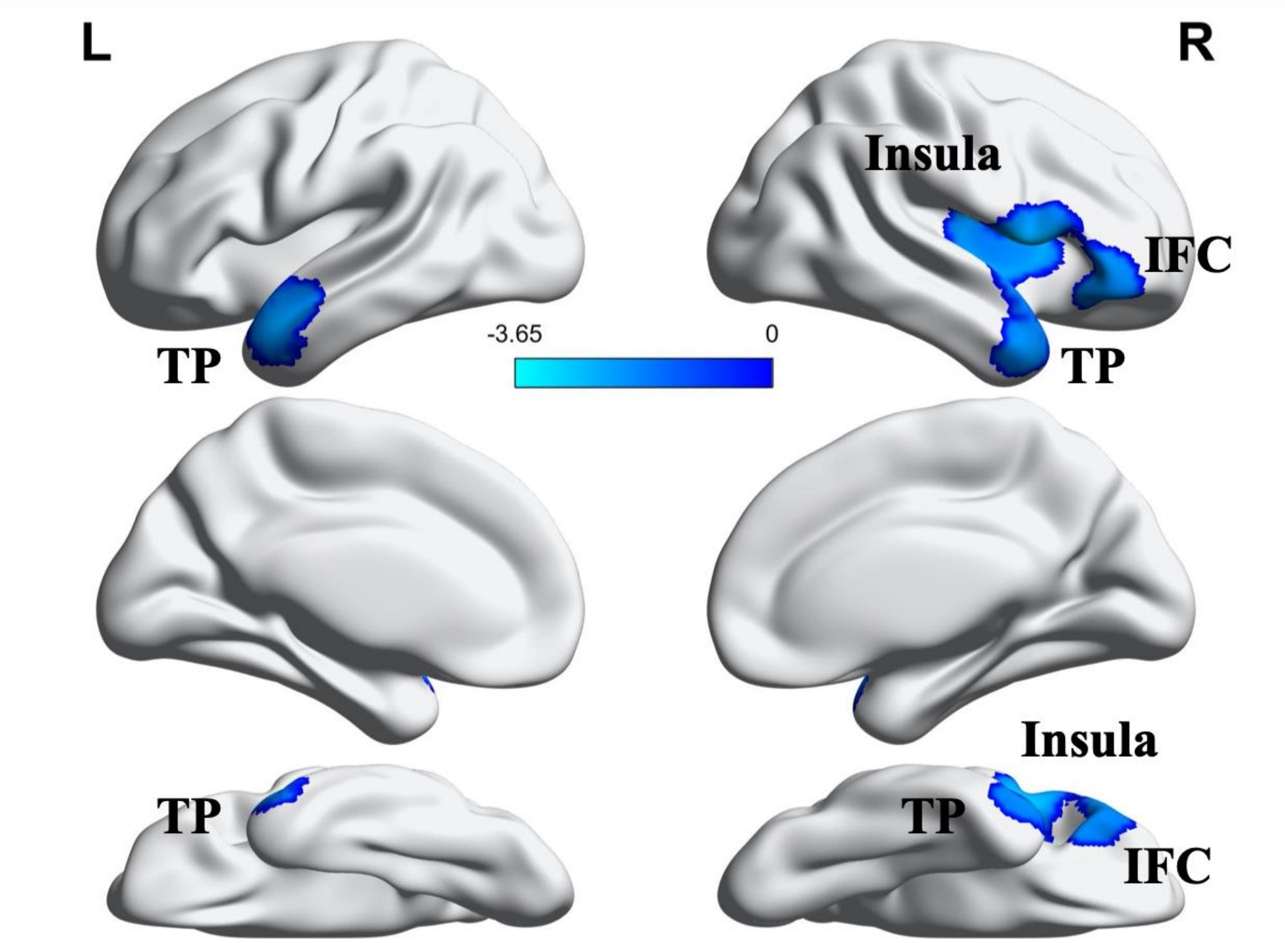

eFigure 4. Meta-regression results in the long-term SCZ group

Meta-regression results show that the illness duration of long-term schizophrenia (SCZ) is negatively correlated with cortical thickness in the left temporal pole (TP). The asterisk (*) indicates that the P value survived the Bonferroni correction.

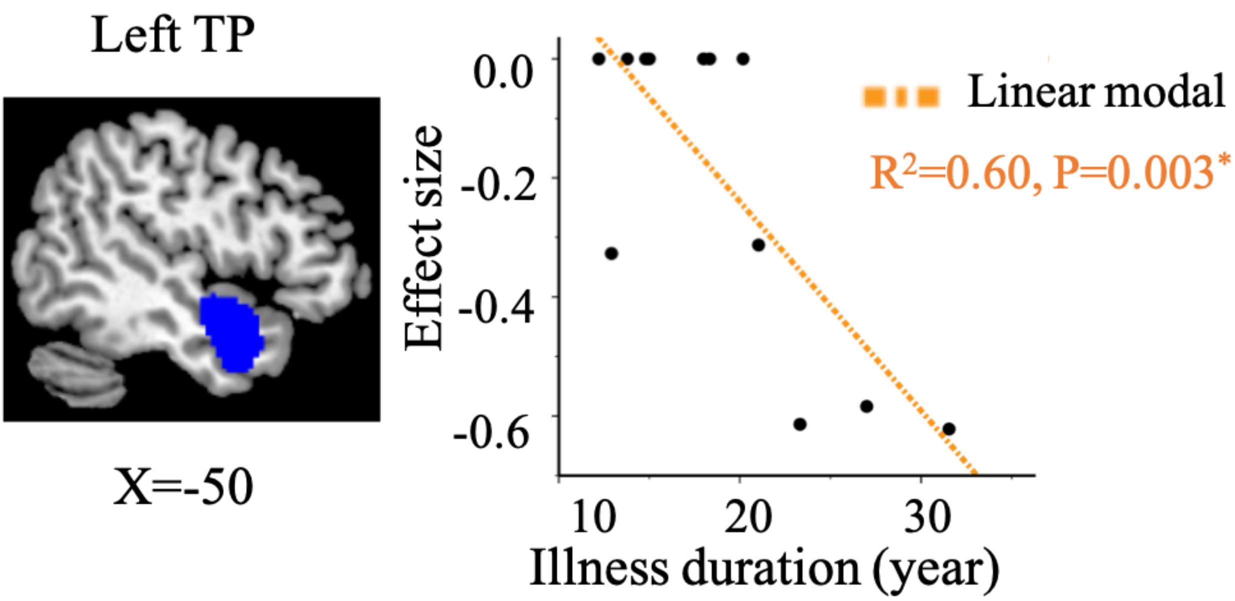

eFigure 5. Differences in cortical thickness between CHR and long-term SCZ groups

Compared with clinical high-risk (CHR) individuals, individuals with long-term schizophrenia (SCZ) showed greater cortical thinning (cool color) in the right insula, right pars orbitalis of inferior frontal cortex (IFC), left lateral temporal cortex (LTC) (including superior temporal cortex (STC) and middle temporal cortex (MTC)), and right temporal pole (TP). Color bar shows the SDM Z values. Abbreviations: L, left; R, right.

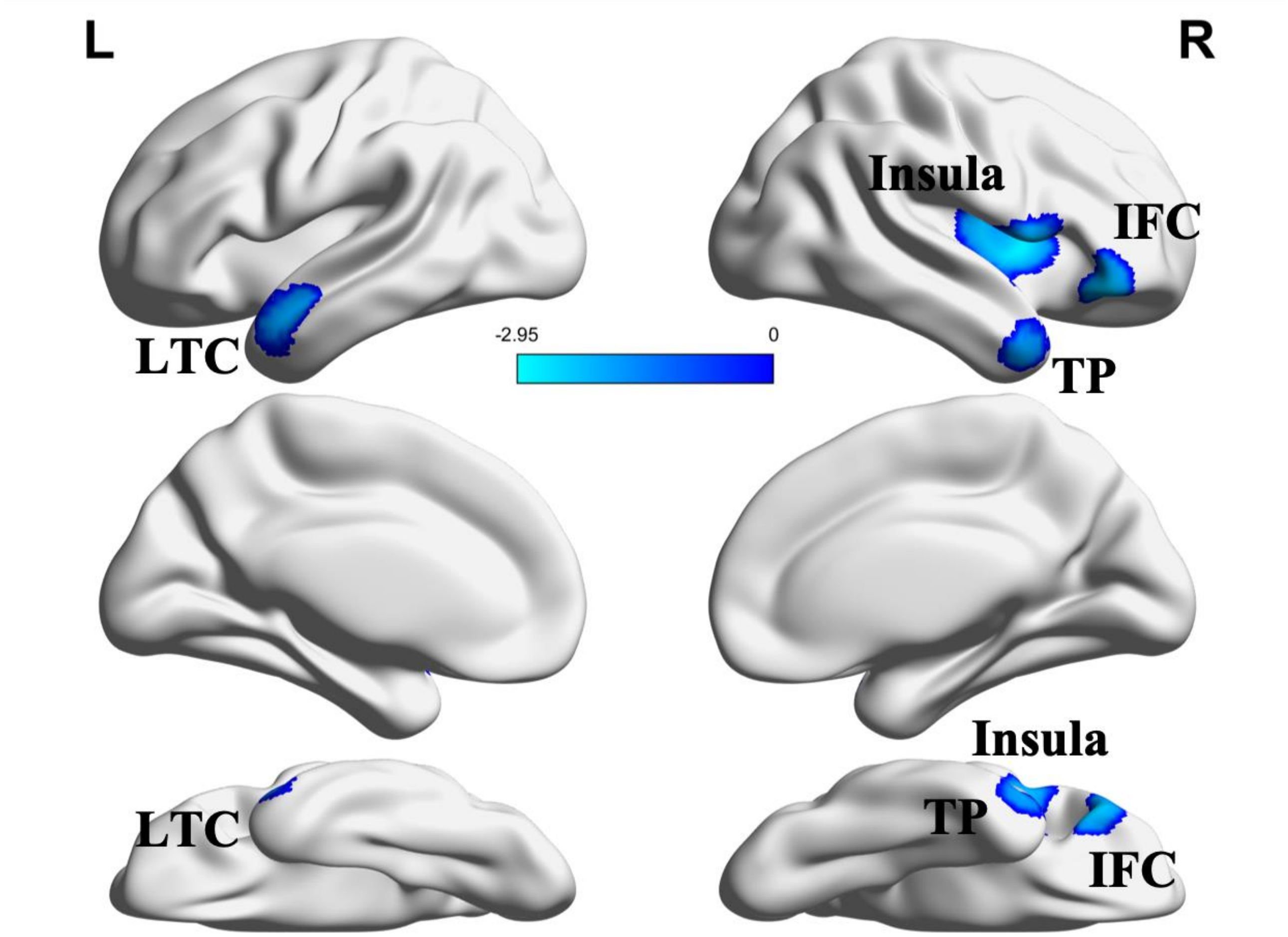

eFigure 6. Differences in cortical thickness between combined group and HCs

CTh reductions (cool color) in the combined group (all studies pooled regardless of illness stage of participants) compared to healthy controls were found in the right insula extending to frontal operculum, left anterior cingulate cortex (ACC) extending to bilateral middle cingulate cortex, right pars orbitalis of inferior frontal cortex (IFC), and bilateral lateral middle temporal cortex (MTC). Abbreviations: Color bar shows the SDM Z values; L, left; R, right.

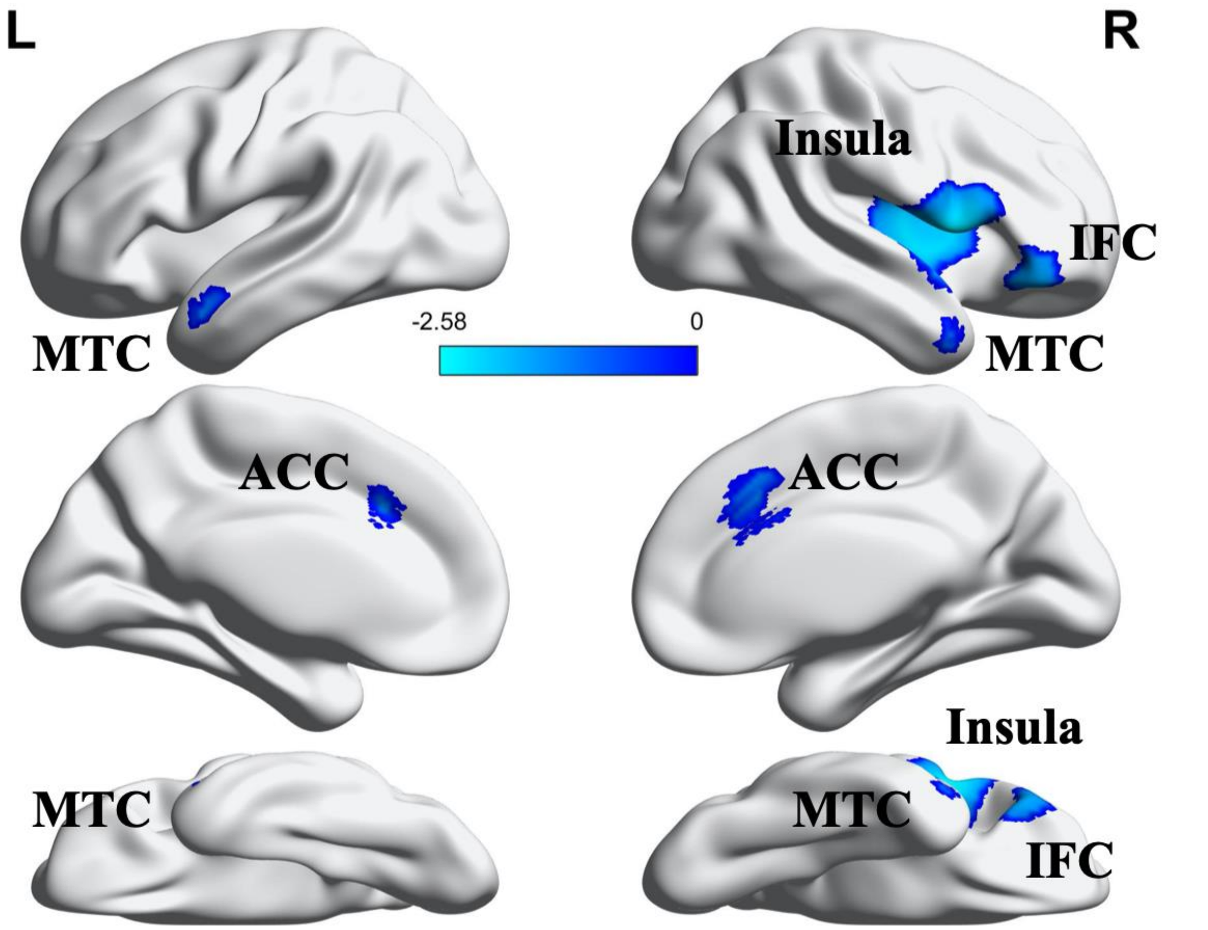

eFigure 7. Meta-regression results in the combined group

Meta-regression results show that the illness duration of the combined group is negatively correlated with cortical thickness in the left lateral middle temporal cortex (MTC). The asterisk (*) indicates that the P value survived the Bonferroni correction.

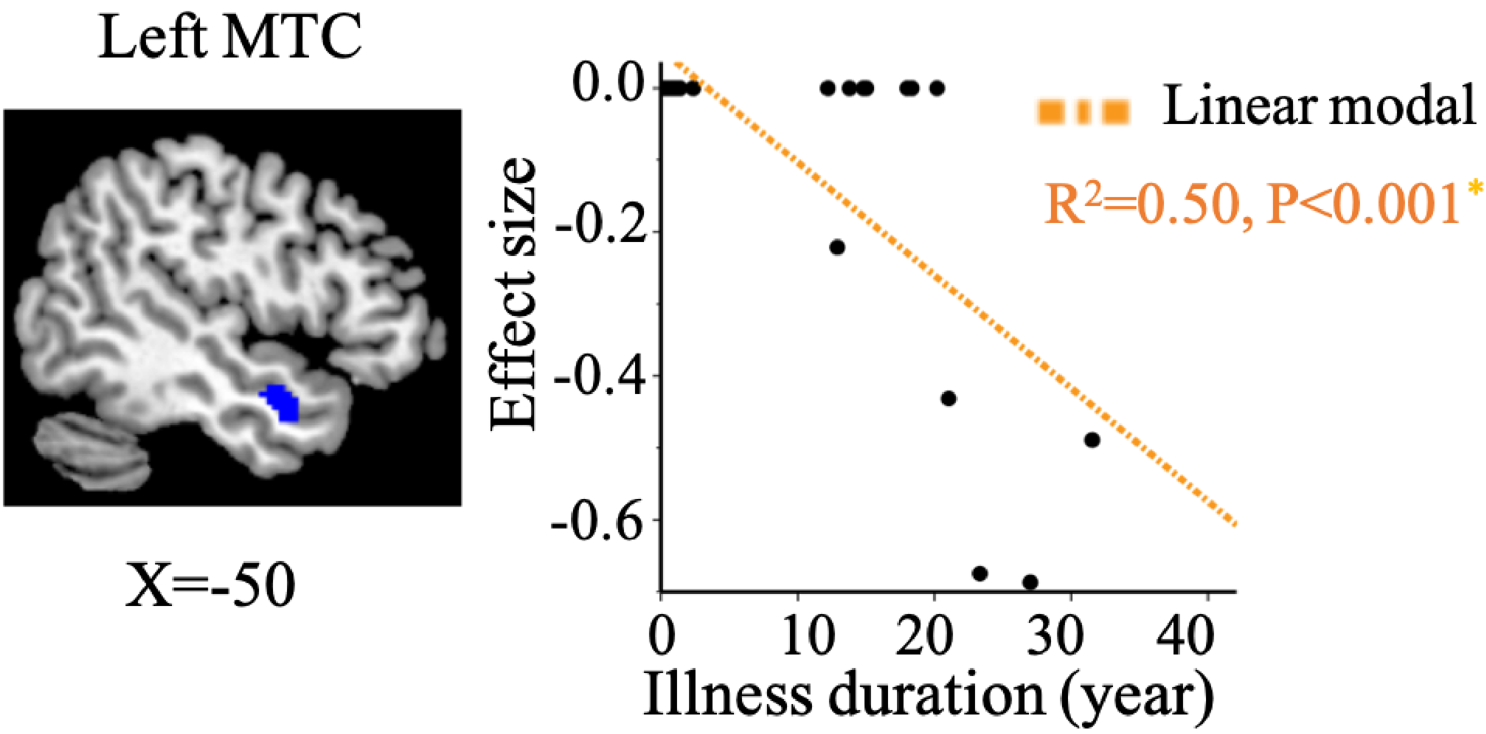

eFigure 8. Results of funnel plot analysis for the meta-analysis of CHR studies

The Egger's test and funnel plots revealed no significant publication bias in the bilateral medial prefrontal cortex (Z=-0.84, t=-1.1, df=11, P=0.31). Abbreviations: CHR, clinical high-risk; SE, standard error.

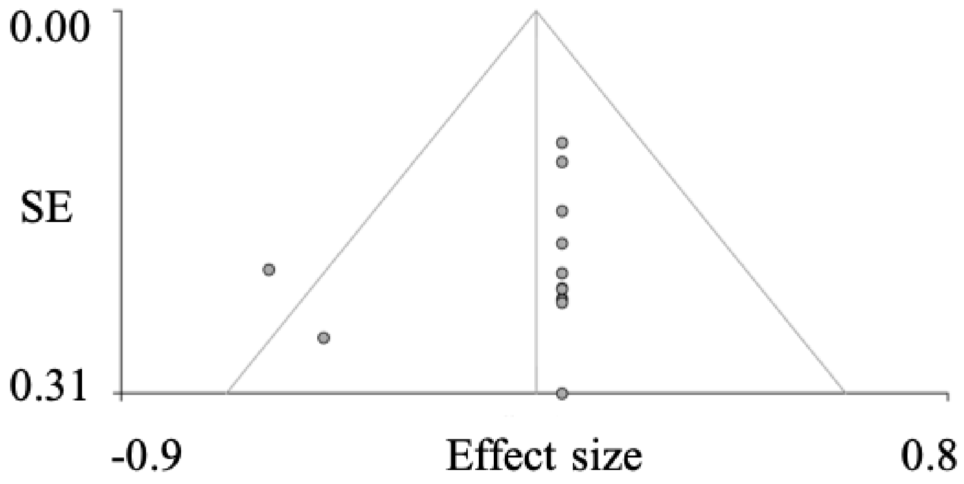

eFigure 9. Results of funnel plot analysis for the meta-analysis of FEP studies

The Egger's test and funnel plots revealed no significant publication bias in the (A) right lateral superior temporal cortex (Z=-0.87, t=-0.81, df=11, P=0.44), (B) right anterior cingulate cortex (Z=-1.96, t=-1.67, df=11, P=0.12), and (C) right insula (Z=-2.12, t=-1.41, df=11, P=0.19). Abbreviations: FEP, first-episode psychosis; SE, standard error.

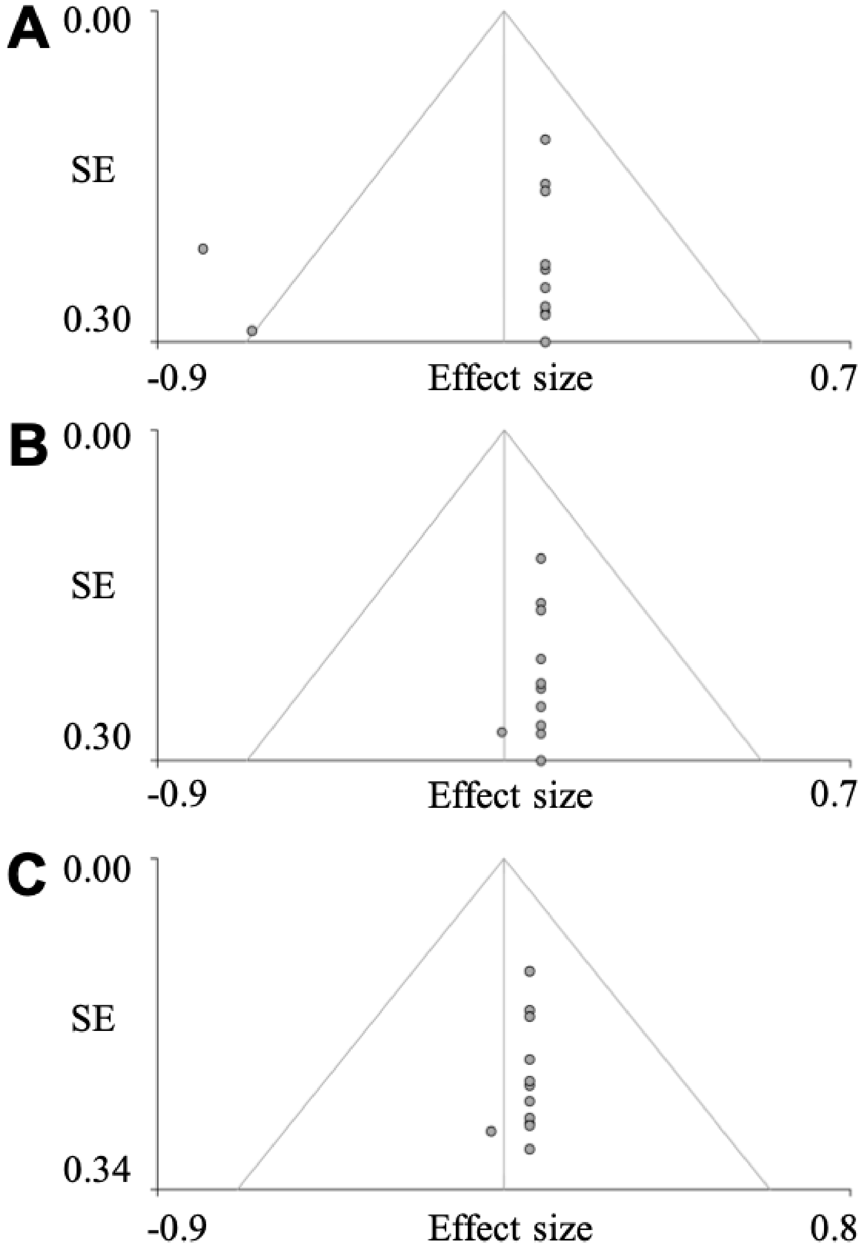

eFigure 10. Results of funnel plot analysis for the meta-analysis of long-term SCZ studies
The Egger's test and funnel plots revealed no significant publication bias in the (A) right insula (Z= 0.77, t=0.70, df=10, P=0.50), (B) right pars orbitalis of inferior frontal cortex (Z=-1.43, t=-1.17, df=10, P=0.27), (C) left temporal pole, middle temporal cortex (Z=-1.72, t=-1.45, df=10, P=0.18), and (D) right temporal pole, superior temporal cortex (Z=-1.01, t=-1.02, df=10, P=0.33). Abbreviations: SCZ, schizophrenia; SE, standard error.

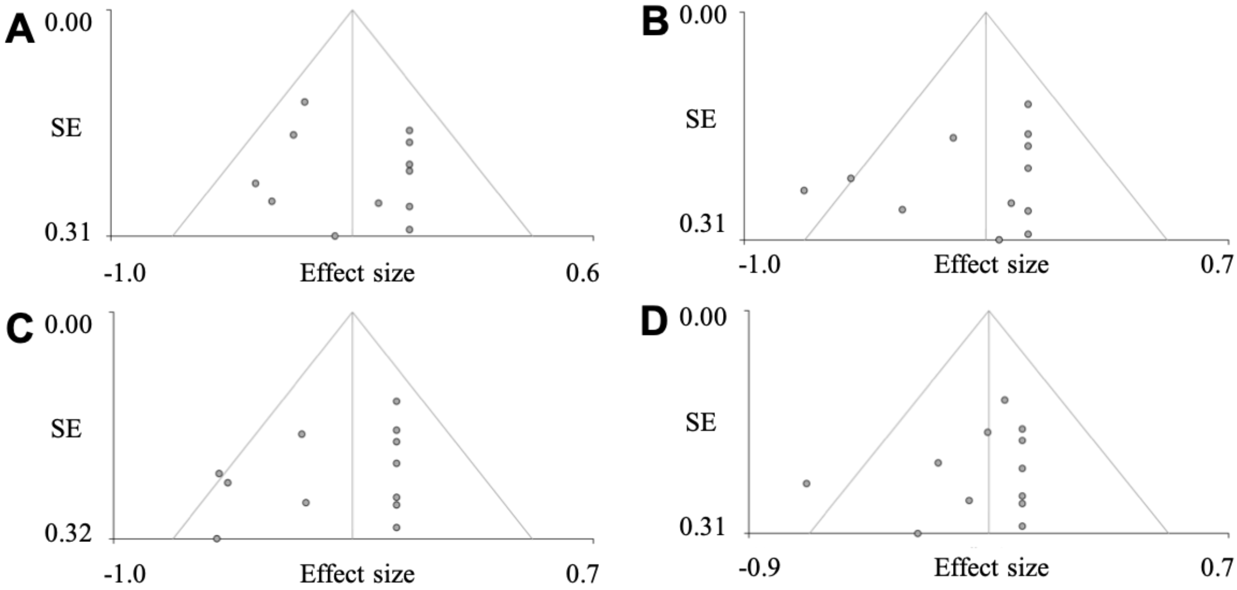

eFigure 11. Results of funnel plot analysis for the pooled meta-analysis of all included studies

The Egger's test and funnel plots revealed no significant publication bias in the (A) right insula (Z=-0.12, t=-0.22, df=36, P=0.83), (B) left anterior cingulate cortex (Z=-0.59, t=-1.52, df=36, P=0.14), (C) right pars orbitalis of inferior frontal cortex (Z=-0.56, t=-0.97, df=36, P=0.34), (D) left lateral middle temporal cortex (Z=-0.66, t=-1.51, df=36, P=0.14), and (E) right lateral middle temporal cortex (Z=-0.40, t=-0.97, df=36, P=0.34). Abbreviations: SE, standard error.

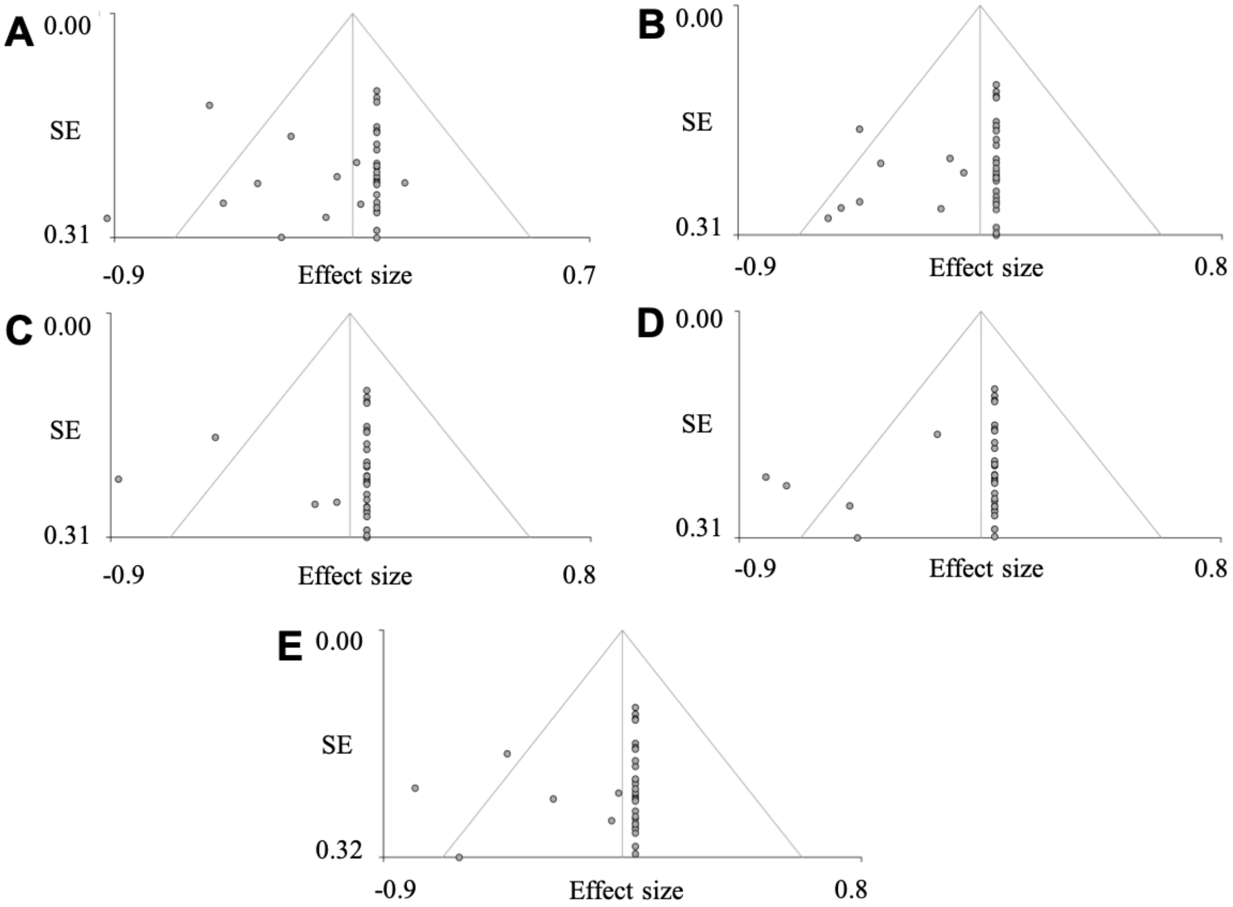

eReferences.

1. Liberati A, Altman DG, Tetzlaff J, et al. The PRISMA statement for reporting systematic reviews and meta-analyses of studies that evaluate health care interventions: explanation and elaboration. *J Clin Epidemiol.* 2009;62(10):e1-34.
2. Du M, Liu J, Chen Z, et al. Brain grey matter volume alterations in late-life depression. *J Psychiatry Neurosci.* 2014;39(6):397-406.
3. Radua J, Mataix-Cols D, Phillips ML, et al. A new meta-analytic method for neuroimaging studies that combines reported peak coordinates and statistical parametric maps. *Eur Psychiatry.* 2012;27(8):605-611.
4. Radua J, van den Heuvel OA, Surguladze S, Mataix-Cols D. Meta-analytical comparison of voxel-based morphometry studies in obsessive-compulsive disorder vs other anxiety disorders. *Arch Gen Psychiatry.* 2010;67(7):701-711.
5. Radua J, Via E, Catani M, Mataix-Cols D. Voxel-based meta-analysis of regional white-matter volume differences in autism spectrum disorder versus healthy controls. *Psychol Med.* 2011;41(7):1539-1550.
6. Li Q, Zhao Y, Chen Z, et al. Meta-analysis of cortical thickness abnormalities in medication-free patients with major depressive disorder. *Neuropsychopharmacology.* 2020;45(4):703-712.
7. Radua J, Mataix-Cols D. Voxel-wise meta-analysis of grey matter changes in obsessive-compulsive disorder. *Br J Psychiatry.* 2009;195(5):393-402.
8. Radua J, Grau M, van den Heuvel OA, et al. Multimodal voxel-based meta-analysis of white matter abnormalities in obsessive-compulsive disorder. *Neuropsychopharmacology.* 2014;39(7):1547-1557.
9. Ziegler G, Dahnke R, Jäncke L, Yotter RA, May A, Gaser C. Brain structural trajectories over the adult lifespan. *Hum Brain Mapp.* 2012;33(10):2377-2389.
10. Zhang W, Deng W, Yao L, et al. Brain Structural Abnormalities in a Group of Never-Medicated Patients With Long-Term Schizophrenia. *Am J Psychiatry.* 2015;172(10):995-1003.

11. Dukart J, Smieskova R, Harrisberger F, et al. Age-related brain structural alterations as an intermediate phenotype of psychosis. *J Psychiatry Neurosci.* 2017;42(5):307-319.

12. Haukvik UK, Hartberg CB, Nerland S, et al. No progressive brain changes during a 1-year follow-up of patients with first-episode psychosis. *Psychol Med.* 2016;46(3):589-598.

13. Reniers RL, Garner B, Phassouliotis C, et al. The relationship between stress, HPA axis functioning and brain structure in first episode psychosis over the first 12 weeks of treatment. *Psychiatry Res.* 2015;231(2):111-119.

14. Cannon TD, Chung Y, He G, et al. Progressive reduction in cortical thickness as psychosis develops: a multisite longitudinal neuroimaging study of youth at elevated clinical risk. *Biol Psychiatry.* 2015;77(2):147-157.

15. Ziermans TB, Schothorst PF, Schnack HG, et al. Progressive structural brain changes during development of psychosis. *Schizophr Bull.* 2012;38(3):519-530.

16. Tognin S, Riecher-Rössler A, Meisenzahl EM, et al. Reduced parahippocampal cortical thickness in subjects at ultra-high risk for psychosis. *Psychol Med.* 2014;44(3):489-498.

17. Buechler R, Wotruba D, Michels L, et al. Cortical Volume Differences in Subjects at Risk for Psychosis Are Driven by Surface Area. *Schizophr Bull.* 2020;46(6):1511-1519.

18. Bakker G, Caan MW, Vingerhoets WA, et al. Cortical Morphology Differences in Subjects at Increased Vulnerability for Developing a Psychotic Disorder: A Comparison between Subjects with Ultra-High Risk and 22q11.2 Deletion Syndrome. *PLoS One.* 2016;11(11):e0159928.

19. Kwak YB, Kim M, Cho KIK, Lee J, Lee TY, Kwon JS. Reduced cortical thickness in subjects at clinical high risk for psychosis and clinical attributes. *Aust N Z J Psychiatry.* 2019;53(3):219-227.

20. Jung WH, Kim JS, Jang JH, et al. Cortical thickness reduction in individuals at ultra-high-risk for psychosis. *Schizophr Bull.* 2011;37(4):839-849.

21. Klauser P, Zhou J, Lim JK, et al. Lack of Evidence for Regional Brain Volume or Cortical Thickness

Abnormalities in Youths at Clinical High Risk for Psychosis: Findings From the Longitudinal Youth at Risk Study. *Schizophr Bull.* 2015;41(6):1285-1293.

22. Gisselgård J, Lebedev AV, Dæhli Kurz K, Joa I, Johannessen JO, Brønnick K. Structural and functional alterations in the brain during working memory in medication-naïve patients at clinical high-risk for psychosis. *PLoS One.* 2018;13(5):e0196289.

23. Buchy L, Makowski C, Malla A, Joober R, Lepage M. A longitudinal study of cognitive insight and cortical thickness in first-episode psychosis. *Schizophr Res.* 2018;193:251-260.

24. Rais M, van Haren NEM, Cahn W, et al. Cannabis use and progressive cortical thickness loss in areas rich in CB1 receptors during the first five years of schizophrenia. *Eur Neuropsychopharmacol.* 2010;20(12):855-865.

25. Ansell BRE, Dwyer DB, Wood SJ, et al. Divergent effects of first-generation and second-generation antipsychotics on cortical thickness in first-episode psychosis. *Psychol Med.* 2015;45(3):515-527.

26. Gutierrez-Galve L, Wheeler-Kingshott CA, Altmann DR, et al. Changes in the frontotemporal cortex and cognitive correlates in first-episode psychosis. *Biol Psychiatry.* 2010;68(1):51-60.

27. Scanlon C, Anderson-Schmidt H, Kilmartin L, et al. Cortical thinning and caudate abnormalities in first episode psychosis and their association with clinical outcome. *Schizophr Res.* 2014;159(1):36-42.

28. Thormodsen R, Rimol LM, Tamnes CK, et al. Age-related cortical thickness differences in adolescents with early-onset schizophrenia compared with healthy adolescents. *Psychiatry Res.* 2013;214(3):190-196.

29. Voets NL, Hough MG, Douaud G, et al. Evidence for abnormalities of cortical development in adolescent-onset schizophrenia. *Neuroimage.* 2008;43(4):665-675.

30. Lesh TA, Tanase C, Geib BR, et al. A multimodal analysis of antipsychotic effects on brain structure and function in first-episode schizophrenia. *JAMA Psychiatry.* 2015;72(3):226-234.

31. Lin Y, Li M, Zhou Y, et al. Age-Related Reduction in Cortical Thickness in First-Episode Treatment-

Naïve Patients with Schizophrenia. *Neurosci Bull.* 2019;35(4):688-696.

32. Nelson EA, Kraguljac NV, White DM, Jindal RD, Shin AL, Lahti AC. A Prospective Longitudinal Investigation of Cortical Thickness and Gyrification in Schizophrenia. *Can J Psychiatry.* 2020;65(6):381-391.

33. Barry EF, Vanes LD, Andrews DS, et al. Mapping cortical surface features in treatment resistant schizophrenia with in vivo structural MRI. *Psychiatry Res.* 2019;274:335-344.

34. Kong L, Herold CJ, Zoellner F, et al. Comparison of grey matter volume and thickness for analysing cortical changes in chronic schizophrenia: A matter of surface area, grey/white matter intensity contrast, and curvature. *Psychiatry Res.* 2015;231(2):176-183.

35. Madre M, Canales-Rodríguez EJ, Fuentes-Claramonte P, et al. Structural abnormality in schizophrenia versus bipolar disorder: A whole brain cortical thickness, surface area, volume and gyrification analyses. *Neuroimage Clin.* 2020;25:102-131.

36. Xie T, Zhang X, Tang X, et al. Mapping Convergent and Divergent Cortical Thinning Patterns in Patients With Deficit and Nondeficit Schizophrenia. *Schizophr Bull.* 2019;45(1):211-221.

37. Quidé Y, Wilhelmi C, Green MJ. Structural brain morphometry associated with theory of mind in bipolar disorder and schizophrenia. *Psych J.* 2020;9(2):234-246.

38. Landin-Romero R, Canales-Rodriguez EJ, Kumfor F, et al. Surface-based brain morphometry and diffusion tensor imaging in schizoaffective disorder. *Am J Psychiatry.* 2017;51(1):42-54.

39. Zugman A, Gadelha A, Assuncao I, et al. Reduced dorso-lateral prefrontal cortex in treatment resistant schizophrenia. *Schizophr Res.* 2013;148(1-3):81-86.

40. Green AE, Croft RJ, Maller JJ, Fitzgerald PB. White matter correlates of episodic memory encoding and retrieval in schizophrenia. *Psychiatry Res Neuroimaging.* 2016;254:188-198.

41. Fortea A, Batalla A, Radua J, et al. Cortical gray matter reduction precedes transition to psychosis in individuals at clinical high-risk for psychosis: A voxel-based meta-analysis. *Schizophr Res.*

2021;232:98-106.

42. Liloia D, Brasso C, Cauda F, et al. Updating and characterizing neuroanatomical markers in high-risk subjects, recently diagnosed and chronic patients with schizophrenia: A revised coordinate-based meta-analysis. *Neurosci Biobehav Rev.* 2021;123:83-103.

43. Hinney B, Walter A, Aghlmandi S, Andreou C, Borgwardt S. Does Hippocampal Volume Predict Transition to Psychosis in a High-Risk Group? A Meta-Analysis. *Front Psychiatry.* 2020;11:614659.

44. Saunders TS, Mondelli V, Cullen AE. Pituitary volume in individuals at elevated risk for psychosis: A systematic review and meta-analysis. *Schizophr Res.* 2019;213:23-31.

45. Smieskova R, Fusar-Poli P, Allen P, et al. Neuroimaging predictors of transition to psychosis--a systematic review and meta-analysis. *Neurosci Biobehav Rev.* 2010;34(8):1207-1222.

46. Wen K, Zhao Y, Gong Q, et al. Cortical thickness abnormalities in patients with first episode psychosis: a meta-analysis of psychoradiologic studies and replication in an independent sample. *Psychoradiology.* 2021;1(4):185-198.

47. Shah C, Zhang W, Xiao Y, et al. Common pattern of gray-matter abnormalities in drug-naive and medicated first-episode schizophrenia: a multimodal meta-analysis. *Psychol Med.* 2017;47(3):401-413.

48. Fraguas D, Díaz-Caneja CM, Pina-Camacho L, Janssen J, Arango C. Progressive brain changes in children and adolescents with early-onset psychosis: A meta-analysis of longitudinal MRI studies. *Schizophr Res.* 2016;173(3):132-139.

49. Olabi B, Ellison-Wright I, Bullmore E, Lawrie SM. Structural brain changes in First Episode Schizophrenia compared with Fronto-Temporal Lobar Degeneration: a meta-analysis. *BMC Psychiatry.* 2012;12:104.

50. Vita A, De Peri L, Deste G, Sacchetti E. Progressive loss of cortical gray matter in schizophrenia: a meta-analysis and meta-regression of longitudinal MRI studies. *Transl Psychiatry.* 2012;2(11):e190.

51. Chan RC, Di X, McAlonan GM, Gong QY. Brain anatomical abnormalities in high-risk individuals,

first-episode, and chronic schizophrenia: an activation likelihood estimation meta-analysis of illness progression. *Schizophr Bull.* 2011;37(1):177-188.

52. Leung M, Cheung C, Yu K, et al. Gray matter in first-episode schizophrenia before and after antipsychotic drug treatment. Anatomical likelihood estimation meta-analyses with sample size weighting. *Schizophr Bull.* 2011;37(1):199-211.

53. Ellison-Wright I, Glahn DC, Laird AR, Thelen SM, Bullmore E. The anatomy of first-episode and chronic schizophrenia: an anatomical likelihood estimation meta-analysis. *Am J Psychiatry.* 2008;165(8):1015-1023.
